# Risk-adapted monitoring is not inferior to extensive on-site monitoring: Results of the ADAMON cluster-randomised study

**DOI:** 10.1177/1740774517724165

**Published:** 2017-08-08

**Authors:** Oana Brosteanu, Gabriele Schwarz, Peggy Houben, Ursula Paulus, Anke Strenge-Hesse, Ulrike Zettelmeyer, Anja Schneider, Dirk Hasenclever

**Affiliations:** 1 Clinical Trial Centre Leipzig, Leipzig University, Leipzig, Germany; 2 Federal Institute for Drugs and Medical Devices, Bonn, Germany; 3 Clinical Trials Centre Cologne, University of Cologne, Cologne, Germany; 4 KKS-Network/National ECRIN Office, University of Cologne, Cologne, Germany; 5 Institute for Medical Informatics, Statistics, and Epidemiology, Leipzig University, Leipzig, Germany

**Keywords:** Risk-adapted monitoring, on-site monitoring, quality management, clinical trial conduct, clinical trial methodology

## Abstract

**Background:**

According to Good Clinical Practice, clinical trials must protect rights and safety of patients and make sure that the trial results are valid and interpretable. Monitoring on-site has an important role in achieving these objectives; it controls trial conduct at trial sites and informs the sponsor on systematic problems. In the past, extensive on-site monitoring with a particular focus on formal source data verification often lost sight of systematic problems in study procedures that endanger Good Clinical Practice objectives. ADAMON is a prospective, stratified, cluster-randomised, controlled study comparing extensive on-site monitoring with risk-adapted monitoring according to a previously published approach.

**Methods:**

In all, 213 sites from 11 academic trials were cluster-randomised between extensive on-site monitoring (104) and risk-adapted monitoring (109). Independent post-trial audits using structured manuals were performed to determine the frequency of major Good Clinical Practice findings at the patient level. The primary outcome measure is the proportion of audited patients with at least one major audit finding. Analysis relies on logistic regression incorporating trial and monitoring arm as fixed effects and site as random effect. The hypothesis was that risk-adapted monitoring is non-inferior to extensive on-site monitoring with a non-inferiority margin of 0.60 (logit scale).

**Results:**

Average number of monitoring visits and time spent on-site was 2.1 and 2.7 times higher in extensive on-site monitoring than in risk-adapted monitoring, respectively. A total of 156 (extensive on-site monitoring: 76; risk-adapted monitoring: 80) sites were audited. In 996 of 1618 audited patients, a total of 2456 major audit findings were documented. Depending on the trial, findings were identified in 18%–99% of the audited patients, with no marked monitoring effect in any of the trials. The estimated monitoring effect is −0.04 on the logit scale with two-sided 95% confidence interval (−0.40; 0.33), demonstrating that risk-adapted monitoring is non-inferior to extensive on-site monitoring. At most, extensive on-site monitoring could reduce the frequency of major Good Clinical Practice findings by 8.2% compared with risk-adapted monitoring.

**Conclusion:**

Compared with risk-adapted monitoring, the potential benefit of extensive on-site monitoring is small relative to overall finding rates, although risk-adapted monitoring requires less than 50% of extensive on-site monitoring resources. Clusters of findings within trials suggest that complicated, overly specific or not properly justified protocol requirements contributed to the overall frequency of findings. Risk-adapted monitoring in only a sample of patients appears sufficient to identify systematic problems in the conduct of clinical trials. Risk-adapted monitoring has a part to play in quality control. However, no monitoring strategy can remedy defects in quality of design. Monitoring should be embedded in a comprehensive quality management approach covering the entire trial lifecycle.

## Introduction

According to Good Clinical Practice (GCP), clinical trials must protect rights and safety of patients and make sure that the trial results are valid and interpretable. Monitoring on-site has an important role in achieving these objectives; it controls trial conduct at trial sites and informs the sponsor on systematic problems. In the past, on-site monitoring with a particular focus on extensive, but non-targeted source data verification often lost sight of systematic problems in trial procedures, thereby endangering GCP objectives.^1,2^ In light of the fact that monitoring is time consuming and generates extensive costs, the efficacy of this expenditure is being questioned more and more.^3,4^ New regulatory guidance, including the recently released addendum to the GCP Guideline (International Council for Harmonisation of Technical Requirements for Pharmaceuticals for Human Use E6 R2),^5^ recommends optimising the efficacy of monitoring and complementing it with other measures to make better use of available resources.^1,2,6^

In 2004, implementation of the European Union’s Clinical Trial Directive 2001/20/EC was a challenge in particular for academic investigator–initiated trials. Frequency and extent of the necessary on-site monitoring were unclear and cost-efficient approaches were urgently needed, but difficult to implement due to a lack of clear guidance. Therefore, we developed a structured approach to perform risk analysis and define corresponding risk-adapted monitoring strategies, published in 2009.^7^

The main idea was to focus monitoring on trial-specific risks to essential GCP objectives, namely, to assure that the rights, integrity and confidentiality of trial subjects are protected and their safety ensured and that data and reported results are reliable. This tool is referenced in the European Medicines Agency’s reflection paper on risk-based quality management^1^ as well as in the US Food and Drug Administration Guidance on a risk-based approach to monitoring.^2^

The Risk ADApted MONitoring (ADAMON) study was set up to investigate whether a trial-specific, risk-adapted, reduced on-site monitoring strategy as proposed in Brosteanu et al.^7^ is as effective as an extensive, non-targeted on-site monitoring strategy in preventing major or critical violation of GCP objectives, as ascertained by independent audits at the end of the trial.

## Methods

### Study design

ADAMON is a stratified, cluster-randomised non-inferiority study. Trial sites within participating clinical trials were randomised either to extensive or to risk-adapted monitoring.^7^ Cluster randomisation was used because monitoring affects trial sites as a whole by retraining local staff concerning trial procedures triggered by detected findings. In addition, applying different monitoring strategies to individual patients within one site was deemed unfeasible.

Inclusion criteria for trials were as follows: randomised, multicentre (at least six trial sites) clinical trials with a non-commercial sponsor; having Standard Operating Procedures for data management and trial supervision, central monitoring of at least basic extent, and classification as K2 (intermediate risk) or K3 (low risk) based on a trial-specific analysis as proposed in Brosteanu et al.^7^ The classification is based on the following components: (a) the potential risk of the therapeutic intervention evaluated in the trial as compared to standard medical care, (b) the presence of at least one of a list of risk indicators for the patient or the trial results and (c) the robustness of trial procedures (reliable and easy to assess primary endpoint, simple trial procedures). A trial belongs to K3 (low risk) if the risk of the therapeutic intervention is comparable to that of standard medical care, no other risk indicators are present and the trial procedures are robust. In contrast, a trial belongs to K1 (high risk) if either the risk of the therapeutic intervention is higher than that of standard medical care, and other risk indicators are present, or if the risk of the therapeutic intervention is markedly higher than that of standard medical care. Trials in monitoring class K1 (high risk) were not included, since in K1 extensive on-site monitoring is only marginally more extensive than risk-adapted monitoring.

Extensive on-site monitoring comprised checking existence of trial subjects, informed consent documents and complete source data verification for all patients. Visits were scheduled as frequently as necessary in order to fulfil the aforementioned monitoring tasks (at least annually while patients in trial).

Risk-adapted on-site monitoring depended on the assigned monitoring class (K2 or K3) and the monitoring findings at the first visit as well as during the trial (for details, see Table 1).

**Table 1.** Risk-adapted on-site monitoring strategy according to the assigned monitoring class (adapted from Brosteanu et al.^7^).

	K2: intermediate		K3: low
*Initiation*	Obligatory		Can be replaced (investigators’ meeting, detailed written instructions)
*First visit*	After the recruitment of 1–2 patients Assessment of the trial site as ‘with’ or ‘without noticeable problems’; a re-evaluation is performed every year^a^		None
*Further visits*	*Trial site with noticeable problems*	*Trial site without noticeable problems*	
*Frequency* Frequency and duration of visits are scheduled on a trial-specific basis	Depending on the site’s recruitment and the catalogue of monitoring tasks (in general at least three times per year)	Depending on the site’s recruitment and the catalogue of monitoring tasks (in general at least one time per year)	One visit at each trial site
*Verification of key data*	Existence and informed consent for all patients Further key data for at least 50% of the site’s patients	Existence and informed consent for all patients Further key data for at least 20% of the site’s patients	For patients recruited so far at the trial site: Existence and informed consent for all patients Further key data for at least 20% of the site’s patients
*Verification of further data*	A 100% source data verification is made for one patient in the site’s random sample (to ascertain any systematic errors)		None
	*Additional ‘for-cause’ visits* if problems or irregularities were found by central monitoring		

a In ADAMON, assessment of the trial site as ‘with’ or ‘without noticeable problems’ followed a process detailed in the trial-specific monitoring manuals. The monitor assessed and documented noticeable problems, taking into account compliance with ICH-GCP and the protocol as well as the resources of the site staff to conduct the trial. This was checked and confirmed by the trial’s project manager.

### Outcomes

Primary endpoint of the ADAMON study is the proportion of audited patients with at least one major or critical violation of essential GCP objectives in one or more of five error domains: informed consent process, patient selection (eligibility criteria critical for safety and/or efficacy), intervention (protocol deviation with impact on patient safety or data validity), endpoint assessment and serious adverse event reporting. Major or critical GCP violations (in the following referred to as ‘major audit findings’) were determined in independent ADAMON audits at the end of the trial looking at all individual patients in all participating trial sites. Auditors were not in contact with monitors and had no vested interest in the trial. The proportions of patients with major audit findings in specific error domains were assessed as secondary endpoints.

For supportive analysis, the proportion of patients with major audit findings that were not already identified by on-site monitoring during the trial was listed as further secondary endpoint. Monitoring findings were extracted retrospectively from monitoring reports. Monitoring reports used templates provided by the respective trial sponsor. However, the supportive analysis turned out to be only feasible for the informed consent process for which monitoring reports included patient-level lists of findings. For other error domains, the different documentation systems for monitoring and audit findings precluded their comparison and reconciliation.

### Measures against bias

Randomisation of trial sites within participating trials was performed centrally in Leipzig stratified by accrual potential (small vs large, if available) and type of site (University Clinic, General Hospital, Surgery, if applicable). The ratio was 1:1, except in one trial 1:2 (extensive:risk-adapted) due to resource limitations. Trial sites were informed by their respective trial sponsor about ADAMON and the planned audits, but not about the assigned monitoring arm.

Audits were standardised using detailed trial-specific audit manuals developed by the ADAMON team. Manuals defined trial-specific protocol requirements to be verified and GCP violations to be counted as major ADAMON audit findings. They counted as audit findings only if they still persisted at the time of auditing. GCP violations remedied by appropriate monitoring follow-up actions were not counted.

Audit findings were documented on an audit case report form separately for each patient of the respective trial site (refer to supplement for a sample audit manual and audit case report form in German). ADAMON audits were performed jointly by teams of two ADAMON-trained auditors. Auditors came from separate institutions and had no prior involvement in the audited trial. Preferably, the same team audited all trial sites of one trial. Audit teams were not informed of the sites’ monitoring strategy and did not have access to any monitoring reports.

Audit findings were reviewed in a blinded manner by members of the ADAMON team and discussed with auditors, as necessary, to ensure that reporting was consistent with the ADAMON audit manuals.

### Procedures

For each screened trial, a structured risk analysis according to Brosteanu et al.^7^ was agreed upon between the ADAMON and the respective trial team, and a monitoring class was determined. In addition, existing procedures for central oversight and data management were discussed. For eligible trials, a contract concerning ADAMON participation was concluded with the trial sponsor.

Key data were defined and trial-specific manuals developed for each monitoring strategy. These manuals were the basis for contracts with the trial sponsors. ADAMON funded extra monitoring costs arising from participation in ADAMON.

Conduct of monitoring was the responsibility of the respective trial sponsor. For each monitoring strategy, disjoint teams of monitors were trained by the ADAMON team. The ADAMON team received the monitoring reports and supervised adherence to the monitoring manuals, providing additional training for monitors if required.

### Statistical analysis

Both the intervention (monitoring strategy) as well as the endpoint assessment (audit) were customised to each trial, but using a common structured approach. Trials varied in complexity. Finding rates are thus only comparable within each trial. ADAMON relies on meta-analysing results obtained within each trial, with a model assuming that trial-specific differences between extensive on-site monitoring and risk-adapted monitoring on the logit scale (i.e. log odds ratios) are comparable across trials.

The protocol-specified analysis is logistic regression incorporating trial and monitoring arm as fixed effects and trial site as a single random effect, corresponding to a fixed-effect meta-analysis. In addition, it assumes that the variance of site random effects can be estimated across trials; such estimates may be unstable with only few sites for a given trial.

As a sensitivity analysis, we also present a standard random-effect meta-analysis of separate and independent trial-specific treatment effects on the logit scale obtained with logistic regression with monitoring arm as fixed effect and site as a random effect.

Primary result of the ADAMON study is an estimate (with 95% confidence interval) of the effect of risk-adapted monitoring on the proportion of patients with major audit findings.

ADAMON set out to recruit 12 trials with at least 100 randomised trial sites accruing at least 3200 patients to achieve a power of 80%. This was based on simulations assuming an average of 10–30 patients included per site, variance of the site random effect of 0.6–1 on the logit scale (describing the cluster effect; corresponding intra-class correlation coefficient between 0.033 and 0.09) and an overall finding rate of 5%–10%.^8^ Evidence for these planning assumptions were limited.

The non-inferiority margin was set to 0.60 on the logit scale. In the planning scenario, this corresponded to an increase in finding rate from 7% with extensive on-site monitoring to 12% with risk-adapted monitoring.

## Results

### Trials

**Figure 1.**
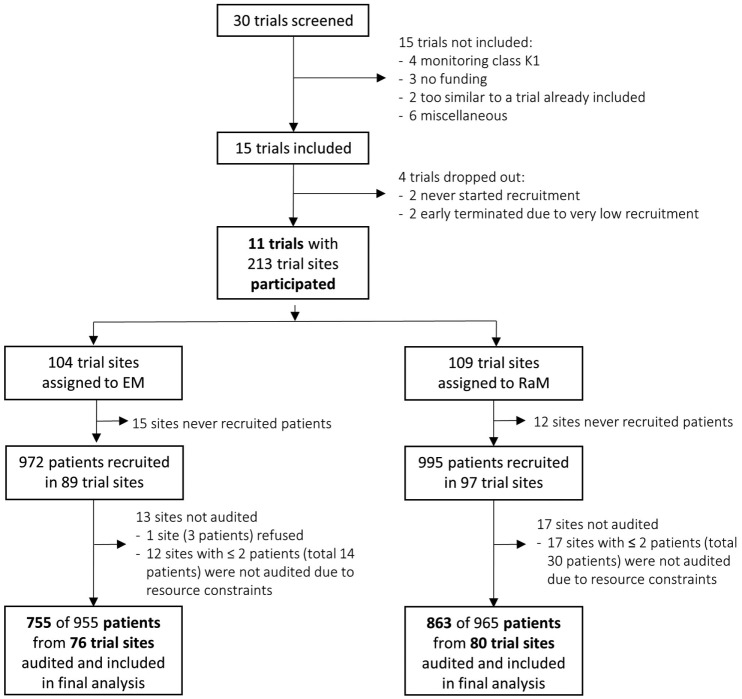
Profile of the ADAMON study. EM: extensive on-site monitoring; RaM: risk-adapted monitoring.

Between April 2009 and June 2012, 30 clinical trials were screened and 15 included. Of those, two never started recruitment, and a further two terminated very early due to insufficient accrual. ADAMON audits were performed in the remaining 11 trials (Figure 1).

Table 2 alphabetically lists and characterises these trials,^9–16^ which cover a broad spectrum of indications. Three trials were assessed as monitoring class K3 (low risk). In 5 of the 11 trials (HYPRESS, HD16, CLL10, TABEA and SYNCHRONOUS, for full trial names see Table 2), only a sample of trial sites took part in ADAMON. In NIC-PD, only the German trial sites were involved. For all further analyses, the 11 trials are pseudonymised using an internal trial number unrelated to the alphabetical order.

**Table 2.** Participating trials in alphabetical order.

Title	Trial identifier	Design	Primary endpoint	Type of case report form	Monitoring class according to Brosteanu et al.^7^
*CeTeG*– Phase III trial of CCNU/temozolomide (TMZ) combination therapy versus standard TMZ therapy for newly diagnosed MGMT-methylated glioblastoma patients	NCT01149109	Multicentre, randomised, open-label, two-arm parallel-group, superiority trial	Overall survival	Paper-based	K2
*CLL10*– Phase III trial of combined immune-chemotherapy with Fludarabine, Cyclophosphamide and Rituximab (FCR) versus Bendamustine and Rituximab (BR) in patients with previously untreated chronic lymphocytic leukaemia^11^	NCT00769522	International, multicentre, randomised, open-label, two-arm parallel-group, non-inferiority trial	Progression-free survival	Paper-based	K2
*HASTA – HA*nd *S*uture *V*ersus *STA*pling for Closure of Loop Ileostomy^14^	DRKS00000040	Multicentre, randomised, open-label, two-arm parallel-group, superiority trial	Rate of bowel obstruction within 30 days after ileostomy closure	Paper-based	K3
*HD16* for early stages – treatment optimisation trial in the first-line treatment of early-stage Hodgkin lymphoma; treatment stratification by means of FDG-PET	NCT00736320	Multicentre, randomised, open-label, two-arm parallel-group, non-inferiority trial	Progression-free survival	Paper-based	K3
*HYPRESS***–** Hydrocortisone for Prevention of Septic Shock^12^	NCT00670254	Multicentre, randomised, double-blind, placebo-controlled, two-arm parallel-group, superiority trial	Septic shock within 14 days	Electronic	K2
*MOOD-HF***–** Effects of selective serotonin re-uptake inhibition on MOrbidity, mOrtality and mood in Depressed Heart Failure patients^9^	ISRCTN33128015	Multicentre, randomised, double-blind, placebo-controlled, two-arm parallel-group, superiority trial	Time to first event of death or hospitalisation	Electronic	K2
*NIC-PD*– A randomised, placebo-controlled, double-blind, multi-centre trial to assess the disease-modifying potential of transdermal nicotine in early Parkinson’s disease in Germany and the USA	NCT01560754	Multicentre, randomised, double-blind, placebo-controlled, two-arm parallel-group, superiority trial	Change of total UPDRS I–III score between baseline and 60 weeks	Electronic	K2
*NINSAPP*– Surfactant Application During Spontaneous Breathing with Continuous Positive Airway Pressure (CPAP) in Premature Infants <27 weeks^13^	ISRCTN64011614	Multicentre, randomised, open-label, two-arm parallel-group, superiority trial	Survival without bronchopulmonary dysplasia at 36 weeks’ gestational age	Paper-based	K2
*ORCHID***–** Open Reduction and Internal Fixation versus Casting for Highly comminuted and Intra-articular Fractures of the Distal Radius^10^	ISRCTN76120052	Multicentre, randomised, open-label, two-arm parallel-group, superiority trial	Short Form 36 Physical Component Score 1 year after the fracture	Paper-based	K3
*SYNCHRONOUS* – Resection of the primary tumour versus no resection prior to systemic therapy in patients with colon cancer and synchronous unresectable metastases (UICC stage IV)^16^	ISRCTN30964555	Multicentre, randomised, open-label, two-arm parallel-group, superiority trial	Overall survival	Electronic	K2
*TABEA***–** A randomised phase III study to determine the efficacy of a taxane and bevacizumab with or without capecitabine as first-line chemotherapy in patients with metastatic breast cancer^15^	NCT01200212	Multicentre, randomised, open-label, two-arm parallel-group, superiority trial	Progression-free survival	Electronic	K2

MGMT: O6-methylguanine-DNA methyltransferase; UICC: Union for International Cancer Control.

For all further analyses, the 11 trials are pseudonymised using an internal trial number unrelated to the alphabetical order.

### Trial sites and audits

Overall, 213 trial sites were randomised between extensive (104) and risk-adapted (109) monitoring. Of the sites, 27 sites never recruited any patient; 186 trials sites, 89 of which were monitored extensively and 97 in a risk-adapted manner, recruited a total of 1967 patients. From these, 30 sites with 47 patients were not audited: one site refused the audit, and in the last five audited trials, 29 sites with less than three patients were not audited due to limited resources (Figure 1). Thus, 156 sites were audited and included in the final analysis: 76 extensively monitored sites, which had enrolled 955 patients, and 80 sites monitored in a risk-adapted manner, which had enrolled 965 patients.

In five trials, audits took place as planned after last patient last visit. Due to funding and time limitations, audits were performed in four trials after last patient in, but before end of trial (mainly trials with long-term follow-up per patient). In two trials, accrual was still ongoing at the time trial sites were audited; in these cases, audits were restricted to patients having completed their treatment.

Files from 1618 of 1967 patients (82.3%) were actually audited. Audit duration was limited to 5 days; thus, in large sites (>45 patients), only a centrally pre-selected random sample of patients was audited. Arms are not fully balanced in numbers of patients audited (755 extensive on-site monitoring and 863 risk-adapted monitoring) overall; this is mainly due to large variance in site sizes. Of the audited patients, 1376 (85.0%) were audited by teams of two or three auditors as planned; 242 patients (15.0%) from small sites were audited by a single auditor after participating in calibrating team audits.

Auditing required 523 auditor days on-site, roughly 2.6 auditor years. Average audit duration per patient was 2.6 h and similar between monitoring arms (2.7 extensive on-site monitoring and 2.5 risk-adapted monitoring).

### Implementation of monitoring

Monitoring efforts differed markedly by monitoring strategy within each participating trial (Figure S1 and Figure S2 supplement). With extensive on-site monitoring, the number of monitoring visits per patient and the cumulative monitoring time on-site was higher compared to risk-adapted monitoring by a factor of 2.1 and 2.7, respectively (ratios of the efforts calculated within each trial and summarised with the geometric mean). As expected, these factors were more pronounced in (low risk) monitoring class K3 (3.5 and 5.2 in K3 vs 1.8 and 2.1 in K2). Average number of visits per site was 5.4 (K3: 4.9; K2: 5.7) with extensive and 2.7 (K3: 1.03; K2: 3.7) with risk-adapted monitoring.

### Audit findings

Overall, 2456 major audit findings were documented in 996 of 1618 (61.6%) audited patients. Table 3 describes trials and their finding rates overall and by monitoring strategy. Overall finding rates differ markedly between participating trials. Patient-level finding rates ranged from 18% to 99%. Broken down by error domain, 241/1618 patients (14.9%) had at least one finding in informed consent process, 331 (20.5%) in patient selection, 405 (25.0%) in intervention, 420 (26.0%) in endpoint assessment and 295 (18.2%) in serious adverse event reporting. Monitoring strategies can only be compared within each trial and site effects have to be accounted for when analysing these raw data.

**Table 3.** Number of sites, patients and patients with any findings overall and by error domain for each trial by monitoring strategy.

Trial	Monitoring strategy	Trial sites	Patients audited	Patients with any major finding		Patients with any major finding in domain IC		Patients with any major finding in domain SEL		Patients with any major finding in domain INTV		Patients with any major finding in domain END		Patients with any major finding in domain SAER	
				N	%	N	%	N	%	N	%	N	%	N	%
#01	EM	6	125	79	63.2	47	37.6	8	6.4	2	1.6	23	18.4	29	23.2
	RaM	5	54	41	75.9	28	51.9	4	7.4	2	3.7	10	18.5	7	13.0
	Overall	11	179	120	67.0	75	41.9	12	6.7	4	2.2	33	18.4	36	20.1
#02	EM	9	107	31	29.0	4	3.7	0	0.0	4	3.7	16	15.0	16	15.0
	RaM	15	212	65	30.7	14	6.6	3	1.4	5	2.4	25	11.8	29	13.7
	Overall	24	319	96	30.1	18	5.6	3	0.9	9	2.8	41	12.9	45	14.1
#03	EM	7	76	57	75.0	39	51.3	10	13.2	7	9.2	11	14.5	22	28.9
	RaM	6	109	86	78.9	40	36.7	31	28.4	16	14.7	12	11.0	33	30.3
	Overall	13	185	143	77.3	79	42.7	41	22.2	23	12.4	23	12.4	55	29.7
#04	EM	9	74	69	93.2	3	4.1	20	27.0	57	77.0	50	67.6	13	17.6
	RaM	7	31	30	96.8	1	3.2	5	16.1	28	90.3	25	80.6	10	32.3
	Overall	16	105	99	94.3	4	3.8	25	23.8	85	81.0	75	71.4	23	21.9
#05	EM	7	35	35	100.0	4	11.4	30	85.7	33	94.3	33	94.3	15	42.9
	RaM	7	33	32	97.0	6	18.2	22	66.7	26	78.8	31	93.9	13	39.4
	Overall	14	68	67	98.5	10	14.7	52	76.5	59	86.8	64	94.1	28	41.2
#06	EM	6	51	14	27.5	2	3.9	2	3.9	0	0.0	11	21.6	2	3.9
	RaM	7	96	47	49.0	16	16.7	3	3.1	7	7.3	24	25.0	12	12.5
	Overall	13	147	61	41.5	18	12.2	5	3.4	7	4.8	35	23.8	14	9.5
#07	EM	6	82	69	84.1	13	15.9	47	57.3	39	47.6	16	19.5	30	36.6
	RaM	5	86	69	80.2	4	4.7	47	54.7	33	38.4	8	9.3	31	36.0
	Overall	11	168	138	82.1	17	10.1	94	56.0	72	42.9	24	14.3	61	36.3
#08	EM	9	93	74	79.6	1	1.1	33	35.5	27	29.0	53	57.0	7	7.5
	RaM	11	99	77	77.8	3	3.0	30	30.3	34	34.3	44	44.4	5	5.1
	Overall	20	192	151	78.6	4	2.1	63	32.8	61	31.8	97	50.5	12	6.3
#09	EM	7	45	10	22.2	4	8.9	4	8.9	4	8.9	1	2.2	0	0.0
	RaM	7	57	8	14.0	3	5.3	2	3.5	2	3.5	2	3.5	1	1.8
	Overall	14	102	18	17.6	7	6.9	6	5.9	6	5.9	3	2.9	1	1.0
#10	EM	7	49	42	85.7	5	10.2	11	22.4	36	73.5	7	14.3	3	6.1
	RaM	8	74	55	74.3	4	5.4	18	24.3	41	55.4	14	18.9	15	20.3
	Overall	15	123	97	78.9	9	7.3	29	23.6	77	62.6	21	17.1	18	14.6
#11	EM	3	18	5	27.8	0	0.0	1	5.6	1	5.6	3	16.7	2	11.1
	RaM	2	12	1	8.3	0	0.0	0	0.0	1	8.3	1	8.3	0	0.0
	Overall	5	30	6	20.0	0	0.0	1	3.3	2	6.7	4	13.3	2	6.7
ADAMON	EM	76	755	485	64.2	122	16.2	166	22.0	210	27.8	224	29.7	139	18.4
	RaM	80	863	511	59.2	119	13.8	165	19.1	195	22.6	196	22.7	156	18.1
	Overall	156	1618	996	61.6	241	14.9	331	20.5	405	25.0	420	26.0	295	18.2

EM: extensive on-site monitoring; RaM: risk-adapted monitoring.

Trials 01, 02 and 08 have low-risk class K3.

Error domains: informed consent process (IC), patient selection (eligibility criteria critical for safety and/or efficacy; SEL), intervention (protocol deviation with impact on patient safety or data validity; INTV), endpoint assessment (END) and serious adverse event reporting (SAER).

The ADAMON protocol specified a generalised linear mixed model, namely, logistic regression with trials and monitoring arm as fixed effects and sites as a single random effect over all trials. The estimated standard deviation of the random effect is 0.64 on the logit scale.

**Figure 2.**
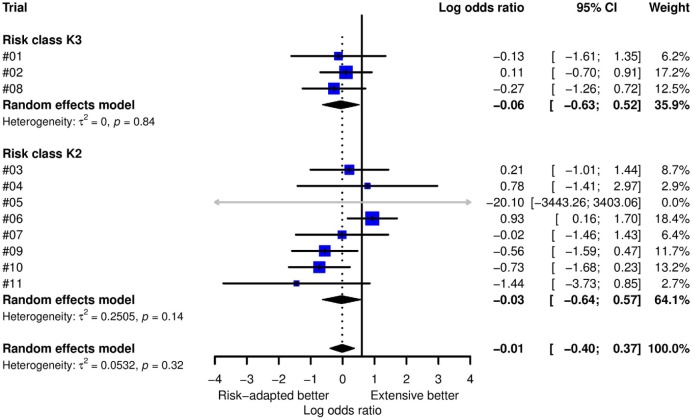
Monitoring effect on the primary endpoint. Figure 2 shows the forest plot of a random-effect meta-analysis of within-trial monitoring effects. The overall estimate of the random-effect meta-analysis closely agrees with the model-based estimate of −0.04 with two-sided 95% confidence interval (−0.40; 0.33). There is no significant heterogeneity between trials. Note that trial #05 is non-informative because there were major findings in all but one patient. Trials are grouped by risk class. The intervention effect does not differ by risk class. The black vertical line at 0.6 shows the pre-specified tolerance margin for claiming non-inferiority. Overall and in both subgroups, the non-inferiority margin is outside the meta-analysis confidence interval (CI).

The patient-level estimate of the monitoring effect is −0.04 on the logit scale with a two-sided 95% confidence interval (−0.40; 0.33). Figure 2 shows the forest plot of a random-effect meta-analysis of within-trial monitoring effects estimated with logistic regression with site as random effect accounting for clustering. There is no significant heterogeneity between trials (estimated heterogeneity variance: 0.05, p = 0.32). Note that trial #05 is statistically non-informative because there were major findings in all but one patient. The intervention effect does not differ by risk class. The overall estimate of the random-effect meta-analysis closely agrees with the model-based estimate.

**Figure 3.**
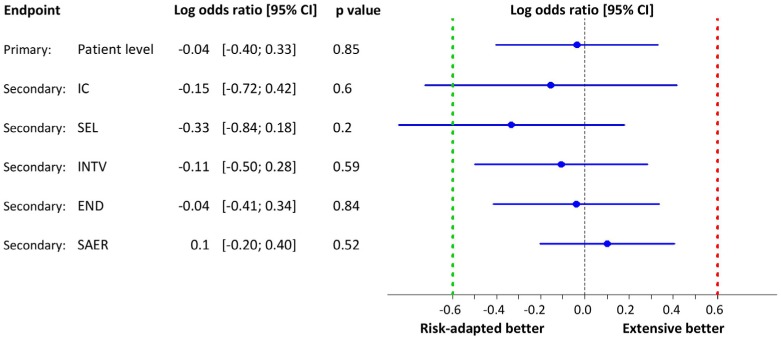
Model-based estimates of the monitoring effect for the primary patient-level and the secondary error domain–specific finding rates. Error domains: informed consent process (IC), patient selection (eligibility criteria critical for safety and/or efficacy; SEL), intervention (protocol deviation with impact on patient safety or data validity; INTV), endpoint assessment (END) and serious adverse event reporting (SAER). There is no statistical evidence that type of monitoring makes any difference in reducing the number of major findings neither overall nor in specific error domains. Quantitatively, point estimates lie near zero on the logit scale and all two-sided 95% confidence intervals clearly exclude the pre-specified tolerance limit of logit +0.6. CI: confidence interval.

Corresponding figures for the error domain–specific finding rates can be found in the supplement Figures S3–S7. Again, random-effect meta-analysis is consistent with the model-based estimates. Figure 3 illustrates model-based estimates of the monitoring effect for the primary patient-level and the secondary error domain–specific finding rates.

There is no statistical evidence that type of monitoring makes any difference in reducing the number of major audit findings either overall or in specific error domains. Quantitatively, point estimates lie near zero on the logit scale, and all two-sided 95% confidence intervals clearly exclude the pre-specified tolerance limit of logit +0.6. Thus, non-inferiority is shown.

### Description of audit findings by error domains

We performed a detailed explorative analysis of all 2456 findings, which will be published separately. The most frequent types of findings by error domain are as follows.

Findings in the informed consent process (N = 292) mainly concerned dating the signature by the participating patient (missing, delayed or not written by patient; N = 180). Another problem was information being provided by staff not qualified for this task (N = 38). We did not find positive evidence that any audited patient entered a trial without being aware of his/her trial participation.

With regard to patient selection (N = 436), measurements required for the assessment of eligibility were not performed, not performed in a timely manner or were out of range in 175 cases. In total, 89 findings concerned violation of complicated rules on prohibited co-medication mainly in one of the trials. In 71 cases, included patients either did not belong to the targeted trial population to which the trial question applied (N = 18) or compliance with eligibility criteria was not fully verifiable.

Findings in intervention (N = 758) mainly concerned treatment modification rules in complex treatment schemes: In 227 findings, a treatment modification rule was ignored, and in further 166, a modification rule trigger (e.g. blood counts before start of next therapy cycle) was not measured or not measured in a timely manner. In all, 90 relevant dose deviations were noted.

A total of 629 findings concerned endpoint assessments: an endpoint was not assessed in 95 cases, measured inadequately in 181 cases and not on schedule in 68 cases.

In serious adverse event reporting (N = 356 findings), the most frequent finding was non-reporting of a serious adverse event (N = 73) or reporting with delay (N = 217). Among these 290 cases where a serious adverse event was not reported or reported with delay, a proportion of 18% (23/128) of serious adverse events were not reported in extensive on-site monitoring compared to 31% (50/162) with risk-adapted monitoring.

### Monitoring findings

Monitoring reported findings in 465 of 755 (61.6%) patients with extensive on-site monitoring and 287 of 863 (33.3%) patients with risk-adapted monitoring. This difference is expected since risk-adapted monitoring required only a sample of patients to be monitored. Finding rates per patient calculated for patients actually monitored in all error domains were comparable (456 of 735 (62.0%) patients with extensive on-site monitoring and 196 of 306 (64.1%) patients with risk-adapted monitoring). This also applies to each error domain (see Figure S8 supplement for a model-based analysis).

### Monitoring findings in relation to subsequent audit findings

The purpose of this section is to report on the quality of monitoring (rate of audit findings not already detected by monitoring) and the degree of remedy of findings through monitoring (rate of monitoring findings not mentioned again by auditors). The informed consent process was both monitored and audited in 1402 cases. Audit and monitoring agreed on ‘no finding’ in 894 and on ‘finding’ in 134 cases, resulting in a concordance rate of 73.3% (extensive on-site monitoring: 73.6%; risk-adapted monitoring: 73.0%). For details, see supplement Table S1. In 76 cases, the audit detected a finding not reported by monitoring (7.8% of all 970 cases without monitoring findings; extensive on-site monitoring: 7.0%; risk-adapted monitoring: 8.7%). Of 432 monitoring findings, 298 (69.0%; extensive on-site monitoring: 65.5%; risk-adapted monitoring: 73.8%) were not reported any more as audit findings, due to monitoring follow-up actions.

## Discussion

ADAMON compared two monitoring strategies: extensive standard on-site monitoring with complete source data verification and less frequent risk-adapted on-site monitoring focussing on key data and trial-specific risks.

The primary endpoint was the number of patients with at least one major or critical violation of GCP objectives as determined by standardised ADAMON post-trial audits. This endpoint was chosen to determine whether the nature and amount of monitoring influence the number of GCP violations that occur in a trial and are not remedied by monitoring.

ADAMON shows that risk-adapted monitoring is non-inferior to extensive monitoring, although it required less than 50% of the monitoring resources.

As trials differed in complexity and therefore overall finding rate, the primary analysis is based on the logit scale. The tolerance margin for the difference in audit finding rates was pre-specified as 0.6 on the logit scale. The estimate of the monitoring effect in the primary endpoint is −0.04 on the logit scale with two-sided 95% confidence interval (−0.40; 0.33). For illustration, Figure S9 (supplement) translates logit differences into easier to interpret finding rate differences depending on an assumed overall finding rate with extensive monitoring. Observed finding rates varied between 18% and 99%. Possible benefit with extensive monitoring not excluded by the conditional 95% confidence intervals is 1.8%, 3.4% and 5.8% for assumed finding rates of 5%, 10% and 20%, respectively. The maximum of 8.2% is attained with finding rates of about 50%. Thus, if there was a benefit from extensive monitoring, the effect would remain small compared to the overall finding rates.

ADAMON only audited 82% of all patients auditable (77.6% with extensive and 86.7% with risk-adapted monitoring). We do not think that this deviation induces bias, since the reasons for not auditing followed the same rules in both arms: We were not able to audit 30 trial sites with less than 3 patients (47 patients in total) due to logistic and financial constraints. In very large trial sites, a random sample of patients was audited, in order to limit auditing to a maximum of 5 days.

The main purpose of monitoring is to help protecting rights and well-being of trial participants and making sure that data are accurate, complete and verifiable from source documents.^5^ Audit findings address these aspects. A secondary objective of monitoring is describing the adherence to GCP and trial rules. Without audit results, knowledge about GCP violations in the trials would clearly depend on the results of monitoring visits only and thus on the monitoring strategy. The absolute number of reported monitoring findings was higher with extensive on-site monitoring than with risk-adapted monitoring, simply because with risk-adapted monitoring only a sample of patients and source data is monitored. But the finding rates per monitored patient were almost identical. This suggests that risk-adapted monitoring provides a representative sample of findings from a sample of patients monitored and thus should be sufficient for the assessment of overall trial protocol compliance and detection of systematic problems in trial conduct, thus allowing the trial sponsor to implement adequate corrective and preventive measures to improve the trial’s quality.

We found some evidence of remedy of GCP violations by monitoring, namely, in informed consent and in a shift from serious adverse event not reported to serious adverse event reported with delay. But remarkably, there was no evidence of an actually preventive effect of monitoring on the occurrence of GCP violations by either monitoring strategy. In particular, there was no consistent trend of less findings in later patients within a trial site (data not shown).

The observed finding rates were surprisingly high (18%–99%). In designing ADAMON, we had assumed a finding rate of about 5%–10% based on a review available at that time.^8^ Finding rates per patient have rarely been reported in a systematic way. ADAMON fills this information gap.

In setting up audit manuals, our aim was to define findings with impact on patient safety and rights and validity of trial results including existence of source data for important trial items. We used strict definitions in ADAMON audit manuals to reduce variance in finding assessment by the auditors. In specifying which deviation from a given rule was to be recorded as a finding, we relied on the wording of trial rules in the respective trial protocols applying rather liberal tolerance limits. Some of the findings can be regarded as a violation of detailed GCP requirements or the wording of a study rule, but not necessarily as a breach of the underlying objective. A detailed attempt to classify ADAMON findings accordingly will be published separately.

In developing the audit manuals from the trial protocols, we were confronted with trial rules and requirements that appeared unnecessarily complex and sometimes ambiguous. Trial protocols also tended to be overly specific causing avoidable friction with local clinical practice. These shortcomings clearly contributed to the high finding rates.

The nature, extent and suspected root causes of the ADAMON audit findings imply that the majority of the GCP violations cannot be retrospectively remedied or prospectively prevented by simple source data verification and subsequent generation of queries. More attention to potential problems would have to be paid while developing the trial protocol and case report forms.

ADAMON had no influence on the quality of design or control of the extent of trial oversight and intensity of central monitoring in the participating trials. Recent recommendations^1,2,17–21^ concerning trial oversight were not available at the time when ADAMON trials started.

ADAMON did not interfere with escalation of and reaction to monitoring findings. Monitoring reports indicate that in some trials, reactions to monitoring findings were unassertive such that systematic problems were not adequately addressed.

ADAMON focussed on academic investigator–initiated trials. We nevertheless hypothesise that results are generalisable beyond academic trials, because error-prone trial rules and error-prone complex clinical settings are not restricted to investigator-initiated trials. This is also supported by publicly available summaries of inspectional observations of the US Food and Drug Administration and the European Medicines Agency.^22,23^

Central statistical monitoring has the potential to detect a considerable proportion of findings without on-site monitoring.^24,25^ ADAMON was not designed to assess how much central statistical monitoring can complement or partly substitute on-site monitoring. In particular, we have not shown that on-site monitoring can be safely omitted.

ADAMON was also not designed to investigate the overall impact of the findings on the reliability of the results of the participating trials. Eight of the trials have been already published, mostly in major journals. Several publications suggest that reliable results can be generated in real-world settings in large trials as long as randomisation and avoidance of systematic bias is guaranteed.^4,3,26^ Two publications^27,28^ suggest that data corrections triggered by source data verification only minimally affect trial outcomes.

To our knowledge, ADAMON is the first fully published trial comparing effectiveness of monitoring strategies;^29–31^ three related studies (OPTI-misation of MONitoring (OPTIMON), Strategic Timing of AntiRetroviral Treatment (START) trial Monitoring Substudy and TargetEd Monitoring: Prospective Evaluation and Refinement (TEMPER) study) – with different foci and designs – will become available in the future. The French OPTIMON study compares the efficacy of two monitoring strategies: one based on the classic standards of quality assurance and the other one based on the risk level (OPTIMON scale) with pre-definition of scientific and regulatory priorities. The study involves clinical research studies with risk level A, B or C (low to intermediate risk) in the OPTIMON scale.^32^ The START monitoring sub-study is part of an international HIV treatment trial. In START, all sites are centrally monitored and are required to perform local quality assurance activities according to a local monitoring plan. In addition, sites are randomised to receive, or not receive, annual on-site monitoring.^33^ Finally, the Targeted Monitoring, Prospective Evaluation and Refinement (TEMPER) study investigates the efficacy of central monitoring in combination with targeted for-cause on-site monitoring. Problematic sites identified by central monitoring are paired with similarly sized inconspicuous sites. Both are monitored on-site. Finding rates are compared using a matched pair design.^34^

Since the design of ADAMON in 2007, the international discussion has moved towards prospective quality by design measures for both the scientific and the operational design of clinical trials and towards the combination of risk-based on-site monitoring with central statistical monitoring.^1–3,17^ ADAMON results provide basic empirical evidence to support this new paradigm, which was scarce up to now.

In conclusion, ADAMON has shown that risk-adapted monitoring is non-inferior to extensive monitoring in avoiding violations of GCP objectives as detected in post-trial audits, although it required less than 50% in monitoring resources. Extensive on-site monitoring compared to risk-adapted monitoring may improve the sponsor’s knowledge about what went wrong in a trial, but does not reduce the number of major or critical GCP violations that occur.

ADAMON results suggest that progress in reducing frequency of GCP violations requires improved quality of trial protocols and comprehensive quality management based on a careful analysis of inherent risks for patients’ safety and rights as well as reliability of trial results.

## Supplementary Material

Supplementary material
