# Supplementary material for: Risk-adapted monitoring is not inferior to extensive on-site monitoring: Results of the ADAMON cluster-randomised study
Source: Clin Trials. 2017 Aug 8;14(6):584–96. doi: 10.1177/1740774517724165 (PMC5718334; doi:10.1177/1740774517724165)
Supplement: Supplementary material [file 724165_ADAMON_Supplement.pdf]

Supplementary material for

Risk adapted monitoring is not inferior to extensive on-site monitoring

Results of the ADAMON cluster-randomised study

Contents

Figure S1: Monitoring visits per patient 3

Figure S2: Monitoring time on-site per patient 4

Figure S3: Monitoring effect on audit findings in error domain Informed Consent Process 5

Figure S4: Monitoring effect on audit findings in error domain Patient Selection 6

Figure S5: Monitoring effect on audit findings in error domain Intervention 7

Figure S6: Monitoring effect on audit findings in error domain Endpoint Assessment 8

Figure S7: Monitoring effect on audit findings in error domain Serious Adverse Event Reporting 9

Figure S8: Monitoring finding rates: model based analysis 10

Table S1: Informed consent process: comparison of monitoring and audit findings 11

Figure S9: Translation of logit differences into finding rate differences 12

ADAMON study protocol 13

Sample Audit Manual and Audit Case Report Forms 13

Figure S1: Monitoring visits per patient

The plot shows the mean number of monitoring visits per recruited patient in audited sites by trial and monitoring strategy. Note that these averages depend on the complexity of the trial and the patient time on trial. They are not comparable across trials.

Trials #01, #02, #08 have low risk class K3; all others are K2.

Extensive on-site monitoring (EM) blue; risk-adapted monitoring (RaM) pink

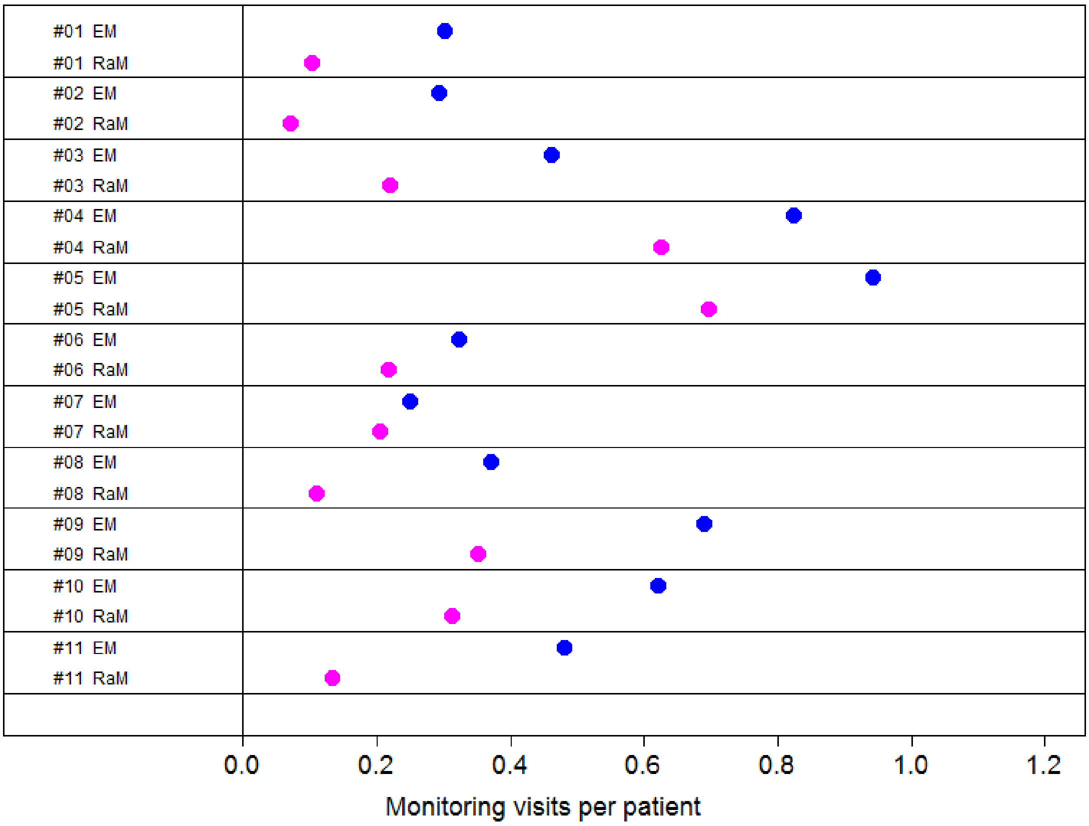

Figure S2: Monitoring time on-site per patient

The plot shows the mean time spent by monitors on-site per recruited patient in audited sites by trial and monitoring strategy. Note that these averages depend on the complexity of the trial and the patient time on trial. They are not comparable across trials.

Trials #01, #02, #08 have low risk class K3; all others are K2.

Extensive on-site monitoring (EM) blue; risk-adapted monitoring (RaM) pink

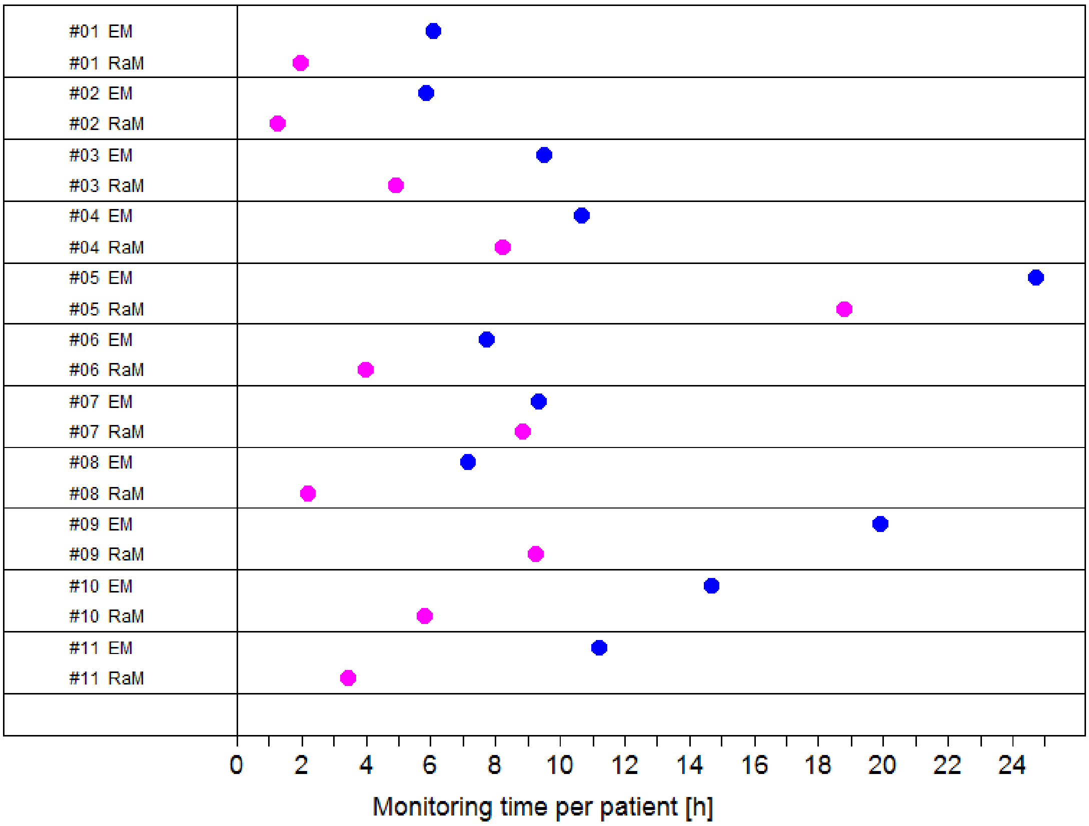

Figure S3: Monitoring effect on audit findings in error domain Informed Consent Process

Figure S3 shows the Forest plot of a random effect meta-analysis of within-trial monitoring effects. The overall estimate of the random effect meta-analysis closely agrees with the model-based estimate of -0.15 with two-sided 95%-confidence interval [-0.72; 0.41]. There is no significant heterogeneity between trials. Note that trial #11 is omitted, because there were no findings at all in the domain Informed Consent Process. Trials are grouped by risk class. The black vertical line at 0.6 shows the pre-specified tolerance margin for claiming non-inferiority. The non-inferiority margin is outside the overall meta-analysis confidence interval (CI).

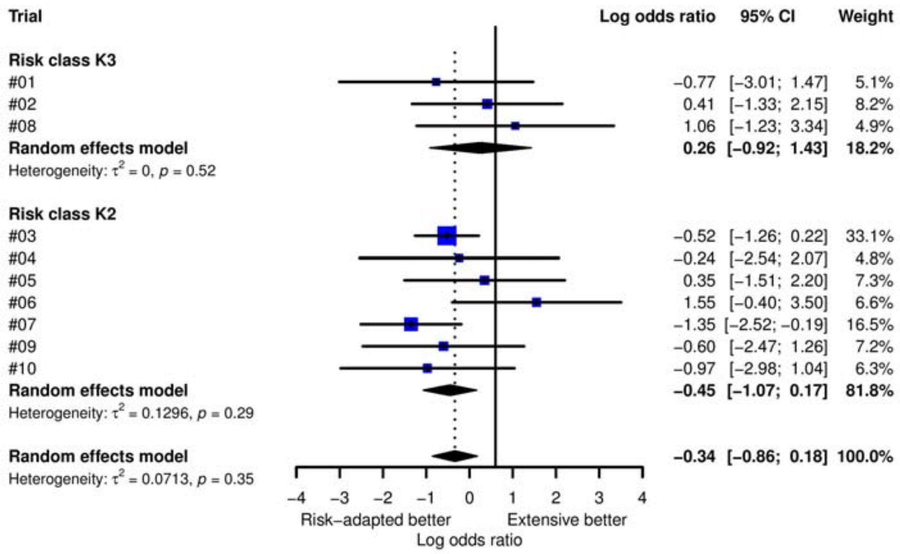

Figure S4: Monitoring effect on audit findings in error domain Patient Selection

Figure S4 shows the Forest plot of a random effect meta-analysis of within-trial monitoring effects. The overall estimate of the random effect meta-analysis closely agrees with the model-based estimate of -0.33 with two-sided 95%-confidence interval [-0.84; 0.18]. There is no significant heterogeneity between trials. Note that trials #02 and #11 are non-informative. In #02 there were only three major findings, none of them with extensive on-site monitoring. In #11there was only one finding (with extensive on-site monitoring). Trials are grouped by risk class. The intervention effect does not differ by risk class. The black vertical line at 0.6 shows the pre-specified tolerance margin for claiming non-inferiority. The non-inferiority margin is outside the overall meta-analysis confidence interval (CI).

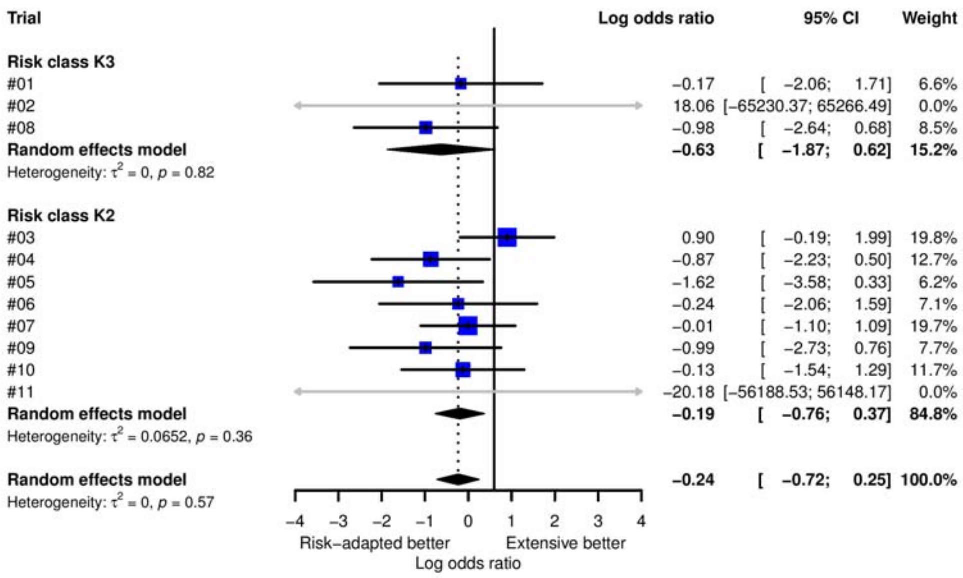

Figure S5: Monitoring effect on audit findings in error domain Intervention

Figure S5 shows the Forest plot of a random effect meta-analysis of within-trial monitoring effects. The overall estimate of the random effect meta-analysis closely agrees with the model-based estimate of -0.04 with two-sided 95%-confidence interval [-0.41; 0.34]. There is no significant heterogeneity between trials. Note that trial #06 is non-informative because there only seven major findings, none of them with extensive on-site monitoring. Trials are grouped by risk class. The black vertical line at 0.6 shows the pre-specified tolerance margin for claiming non-inferiority. The non-inferiority margin is outside the overall meta-analysis confidence interval (CI).

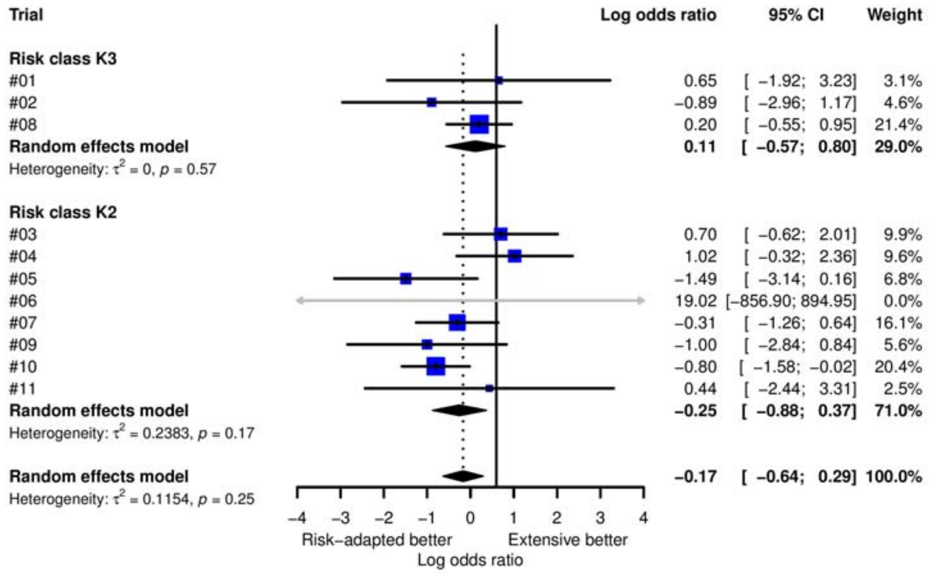

Figure S6: Monitoring effect on audit findings in error domain Endpoint Assessment

Figure S6 shows the Forest plot of a random effect meta-analysis of within-trial monitoring effects. The overall estimate of the random effect meta-analysis closely agrees with the model-based estimate of -0.11 with two-sided 95%-confidence interval [-0.50; 0.28]. There is no significant heterogeneity between trials. Trials are grouped by risk class. The black vertical line at 0.6 shows the pre-specified tolerance margin for claiming non-inferiority. Overall and in both subgroups, the non-inferiority margin is outside the meta-analysis confidence interval (CI).

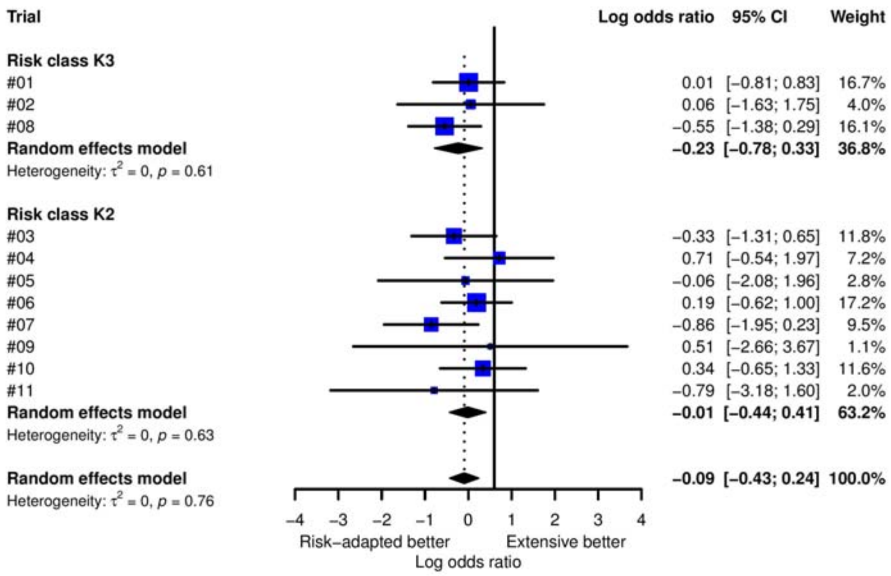

Figure S7: Monitoring effect on audit findings in error domain Serious Adverse Event Reporting

Figure S7 shows the Forest plot of a random effect meta-analysis of within-trial monitoring effects. The overall estimate of the random effect meta-analysis closely agrees with the model-based estimate of -0.1 with two-sided 95%-confidence interval [-0.20; 0.40]. There is no significant heterogeneity between trials. Note that trials #09 and #11 are non-informative. In #09, there was only one finding (with risk-adapted monitoring). In #11, there were two findings, both with extensive on-site monitoring. Trials are grouped by risk class. The black vertical line at 0.6 shows the pre-specified tolerance margin for claiming non-inferiority. The non-inferiority margin is outside the overall meta-analysis confidence interval (CI).

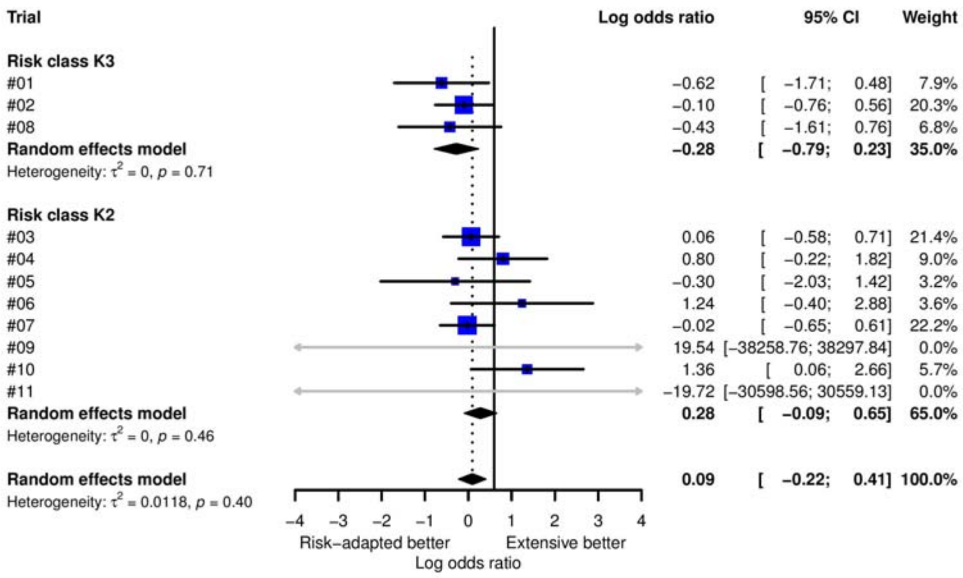

Figure S8: Monitoring finding rates: model based analysis

Figure S8 shows the model-based estimates of the monitoring effect for the patient-level and the error domain specific monitoring finding rates in patients actually monitored. The model-based estimates are all close to zero on the logit scale. Thus, patient-level as well as error domain specific monitoring finding rates are comparable for both monitoring strategies.

Error domains: Informed consent process (IC), Patient selection (eligibility criteria critical for safety and/or efficacy) (SEL), Intervention (protocol deviation with impact on patient safety or data validity) (INTV), Endpoint assessment (END), Serious adverse event reporting (SAER).

EM: extensive on-site monitoring, RaM: risk-adapted monitoring

CI: confidence interval

Monitoring finding	Log odds ratio (95% CI)	p value
Patient level	0.13 (-0.41; 0.67)	0.64
IC	0.09 (-0.44; 0.62)	0.74
SEL	-0.25 (-0.90; 0.40)	0.45
INTV	-0.06 (-0.76; 0.63)	0.86
END	-0.03 (-0.57; 0.52)	0.92
SAER	-0.11 (-0.63; 0.40)	0.66

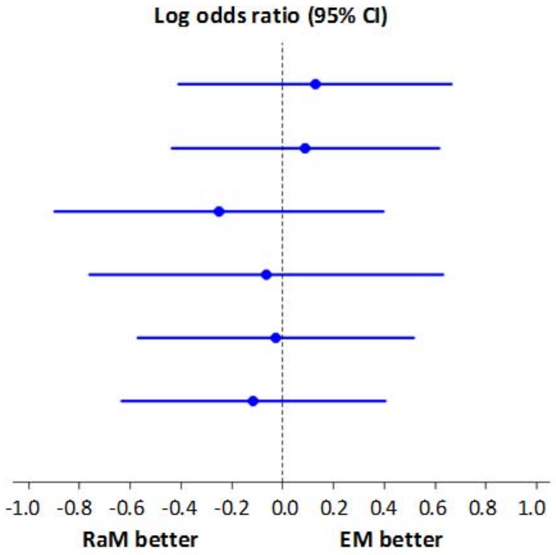

Table S1: Informed consent process: comparison of monitoring and audit findings

In 1402 cases, the informed consent process was both monitored and audited.

In 894 cases, monitoring and audit agreed on “no finding”. In 134 cases, monitoring and audit agreed on “finding”. The resulting concordance rate is 73.3% (1027 / 1402).

In 298 of 432 cases (69.0%), an initially reported monitoring finding was not counted as audit finding any more. This is due to monitoring follow-up actions, which were documented in the monitoring reports.

In 76 of 970 (7.8%) cases, the audit detected a finding, though monitoring reports indicated “no finding”.

		**Audit finding**		
		No	Yes	Total
**Monitoring finding**	No	894	76	970
	Yes	298	134	432
	Total	1192	210	1402

A: All patients

		**Audit finding**		
		No	Yes	Total
**Monitoring finding**	No	465	35	500
	Yes	163	86	249
	Total	628	121	749

B: Patients monitored with extensive on-site monitoring

		**Audit finding**		
		No	Yes	Total
**Monitoring finding**	No	429	41	470
	Yes	135	48	183
	Total	564	89	653

C: Patients monitored with risk-adapted monitoring

Figure S9: Translation of logit differences into finding rate differences

Figure S9 shows conditional confidence intervals for finding rate differences as a function of an assumed finding rate with extensive monitoring. These confidence intervals were derived from the logit scale confidence interval of the primary endpoint. The red curve represents the pre-specified tolerance margin.

Observed finding rates varied between 18 and 99%. Possible benefit with extensive monitoring not excluded by the conditional 95% confidence interval results attains a maximum of 8.2% with finding rates of about 50%.

EM: extensive on-site monitoring, RaM: risk-adapted monitoring

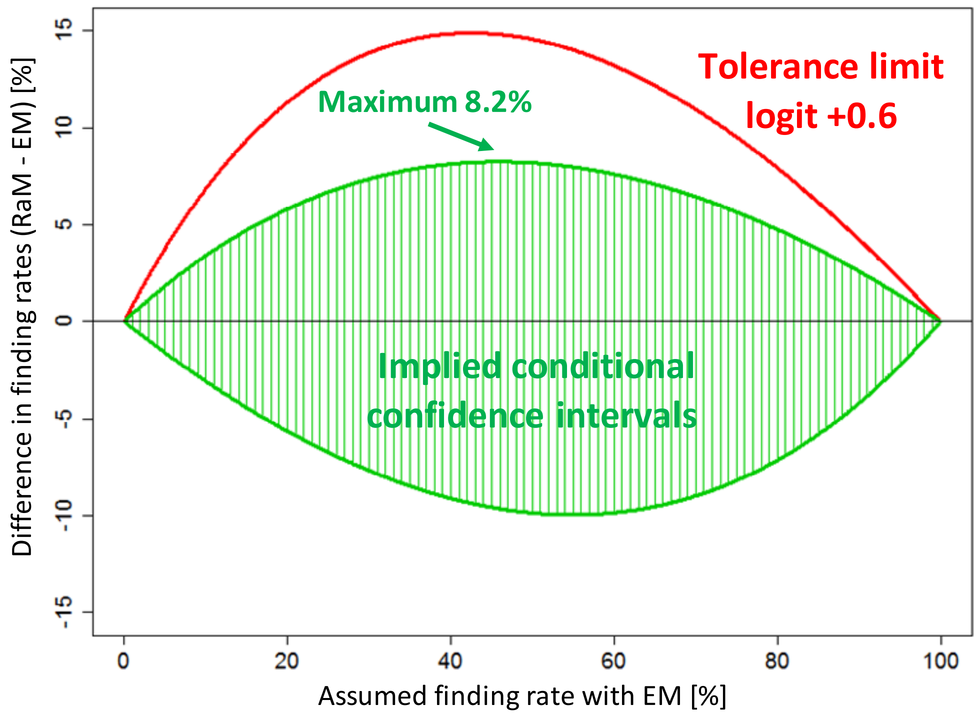

The following table describes the excluded and the tolerated (log odds=0.6) differences in finding rates for specified finding rates with extensive monitoring.

Assumed finding rate with EM	Excluded rate difference	Tolerated difference (0.6 on logit scale)
5%	1.8%	3.8%
7%	2.3%	4.9%
10%	3.4%	6.8%
15%	4.7%	9.3%
20%	5.8%	11.3%
30%	7.3%	13.8%
40%	8.1%	14.8%
50%	8.2%	14.6%
55%	7.9%	13.9%

ADAMON study protocol

The full study protocol (in German) can be accessed via www.adamon.de.

Sample Audit Manual and Audit Case Report Forms

Audit manuals and audit case report forms were set up in German. However, we decided to present a sample in this web appendix, in order to allow an impression on the structure and extent of these documents.

ADAMON
ADAPTIERTES MONITORING

AUDIT MANUAL

HYPRESS-Studie

**Studienname:**	Hydrocortison zur Prävention des septischen Schocks	
**Studiennummer:**	Projekt Nr.	01KG0701
**Audit Manual:**	Version:	Final 3.0
	Datum:	25.08.2014

**Person/Funktion**	**Name**	**Datum**	**Unterschrift**
Autor<br>**ADAMON Projektmanager**	A. Schneider		
Geprüft und genehmigt<br>**ADAMON Projektleitung**	Dr. O. Brosteanu		

**Der Auditor verpflichtet sich, keine Versuche zu unternehmen, die Monitoring-Strategie des auditierten Zentrums herauszufinden, wie etwa durch Review des ISF, Nachfragen oder ähnliches.**			
Gelesen<br>***Auditor***	S. Koch		
Gelesen<br>***Auditor***	M. Vissiennon		

INHALTSVERZEICHNIS

1 ZIEL DER AUDITS 3
2 ALLGEMEINES 3
2.1 Sponsor und Auftragnehmer 3
2.2 Projektmanagement 3
2.3 Kommunikation 3
3 INFORMATIONEN ZUR STUDIENDOKUMENTATION 3
3.1 Audit Unterlagen 3
3.2 Dokumentation und Datenmanagement 4
3.3 Patientenaufklärung und –einwilligung 4
3.4 Behandlung von vorzeitigen Studienabbrüchen 5
3.5 Patientennummer – Patienten-ID 5
3.6 Fehlende Werte 5
3.7 Datenkorrekturen 5
4 INFORMATIONEN ZUM MONITORING 5
5 AUDITING 5
5.1 Umfang des Audits 5
5.2 Vorbereitung der Audits 6
5.3 Prüfarztordner (Investigator Site File, ISF) 6
5.4 Einschätzung des Prüfzentrums 7
5.5 Quelldokumente 7
5.6 Verstöße gegen die Ziele der GCP 7
5.6.1 Mängel bei Aufklärungsprozess und Einwilligungserklärung/Datenschutzerklärung 9
5.6.2 Mängel bei Ein- und Ausschlusskriterien 13
5.6.3 Mängel bei prüfplangemäßer Therapie im Studienverlauf 15
5.6.4 Mängel bei Bestimmung des primären Endpunktes 16
5.6.5 Mängel bei Bestimmung der sekundären Endpunkte 17
5.6.6 Mängel bei der Erfassung und Meldung unerwünschter Ereignisse 18
5.6.7 Mängel bei der Nachbeobachtung 21
5.7 Erstellen der Auditberichte 21
6 ABKÜRZUNGSVERZEICHNIS 21
7 ANHANG 21

1 Ziel der Audits

Die in diesem Audit-Manual beschriebenen Audits erfolgen im Rahmen des ADAMON-Projekts.

Das ADAMON-Projekt ist eine prospektive, cluster-randomisierte Untersuchung studienspezifisch adaptierter Strategien für das Monitoring vor Ort. Ziel des ADAMON-Projektes ist es zu untersuchen, ob eine studienspezifisch adaptierte, reduzierte on-site Monitoringstrategie im Vergleich zu einem umfangreichen vollen Monitoring nach kommerziellen Standards gleichwertig ist. Zielgröße ist hierfür das Auftreten von schwerwiegenden oder kritischen Mängeln (festgestellt im Rahmen eines abschließenden Audits). Es wird eine prospektive, randomisierte Untersuchung durchgeführt. Innerhalb mehrerer klinischer Studien wird für jedes teilnehmende Prüfzentrum randomisiert entschieden, ob das Zentrum mit einer spezifisch adaptierten oder mit einer intensiven Monitoring-Strategie vor Ort überwacht wird. Für jedes Prüfzentrum ist ein abschließendes Audit geplant, im Rahmen dessen schwerwiegende Verstöße gegen die GCP-Guideline erfasst werden. Die Audit-Ergebnisse sind für den Vergleich der beiden Monitoring-Strategien entscheidend.

Hinweis: Während der Studiendurchführung trat das 2. Gesetz zur Änderung arzneimittelrechtlicher und anderer Vorschriften in Kraft (Oktober 2012), deren prüferrelevanten Änderungen (Änderung der Prüferdefinition) in der Hypress-Studie umgesetzt wurden. Dies ist beim Audit hinsichtlich der Aufklärung der Patienten und Meldung von SAEs zu beachten.

2 Allgemeines

2.1 Sponsor und Auftragnehmer

Bei der vorliegenden klinischen Prüfung handelt es sich um eine Studie nach dem deutschen Arzneimittelgesetz. Sponsor der klinischen Prüfung nach §4 Abs. 23 AMG ist die Charité Universitätsmedizin Berlin.

Die Hypress-Studie wurde national mit 380 Patienten und 38 Prüfzentren geplant. In das ADAMON Projekt wurden davon 22 Prüfzentren mit 200 geplanten Patienten eingeschlossen, von denen 12 Prüfzentren aktiv Patienten rekrutierten.

Der Leiter der klinischen Prüfung der Hypress-Studie ist Prof. Dr. Konrad Reinhart (Universität Jena). Die Sponsoraufgaben wurden per Vertrag an PD Dr. Didier Keh - bevollmächtigter Vertreter des Sponsors - übertragen. PD Dr. Keh ist ebenfalls wissenschaftlicher Koordinator der Hypress-Studie. Das ZKS Leipzig - KKS übernimmt für den Sponsor u. a. die Sicherstellung eines angemessenen klinischen Monitorings.

2.2 Projektmanagement

Das Projektmanagement erfolgt durch das ZKS Leipzig – KKS.

2.3 Kommunikation

Primäre Ansprechpartner für die Auditoren sind die Mitarbeiter des ADAMON-Projektes. Etwaige Fragen klinisch/medizinischer oder biometrischer Natur gehen an die Mitarbeiter des ADAMON-Projekts. Es erfolgt keine Kontaktaufnahme der Auditoren zur Studienleitung oder Biometrie.

3 Informationen zur Studiendokumentation

3.1 Audit Unterlagen

Jeder Auditor erhält zur Vorbereitung des Audits alle erforderlichen Dokumente in elektronischer Form (siehe Tabelle).

Dokumentenart	*Dokument, ggf. Version/Datum*	*Inhalt der Änderungen bezogen auf Vorversion bzw. Kommentare*
Prüfplan mit Amendment 1	*HYPRESS_Prüfplan_WorkingVersion (Amendment 01 enthalten)*	Nicht zutreffend, da Rekrutierungsbeginn erst nach Amendment 1
Patienteninformation, Einwilligungserklärung, Datenschutzerklärung	*HYPRESS_Information-Einwilligung_Patient_v2.0_2008-08-19-final*	*Rekrutierungsbeginn mit finaler Version 2.0*
	HYPRESS_Information-Einwilligung_Betreuer_v2.0_2008-08-19-final	Rekrutierungsbeginn mit finaler Version 2.0
	HYPRESS_Information-Einwilligung_Bevollmächtigter_v2.0_2008-08-19-final	Rekrutierungsbeginn mit finaler Version 2.0
	07d_HYPRESS_Erklärung Konsiliar_v2.0_2008-08-19-final	Rekrutierungsbeginn mit finaler Version 2.0
CRF	*HYPRESS_CRF_final_4.0_mit_AE_2013-04-19*	*Rekrutierungsbeginn mit finaler Version 2.0 – relevant für Audit ist nur die Version 4.0, da Änderungen seit Version 2.0 alle Patienten betreffen*
Formblatt SAE Report	*HYPRESS_SAE_Bogen_final2.0_engl_2008-11-11*	*Rekrutierungsbeginn mit finaler Version 2.0*
Personal- und Delegierungsliste (PDL)	*Personal- und Delegierungsliste_final1.0_2008-11-14*<br>*→ zutreffend für alle Prüfzentren*	
	Personal- und Delegierungsliste_final4.0_2012-11-22<br>*→ nur zutreffend für folgende Prüfzentren / betroffene Personen:*<br>*04 Berlin: Stellvertreter, ärztliche Mitarbeiter*<br>*44 Oldenburg: Stellvertreter*<br>*33 Jena: ärztliche Mitarbeiter*	*Funktionen und Aufgaben der Mitarbeiter der Prüfgruppe aktualisiert entsprechend dem*<br>*2. AMG-Änderungsgesetz und damit verbundene Änderung der GCP-Verordnung*<br>*Vorgehen im Prüfzentrum: bisherige PDL final1.0, beendet, neue PDL final4.0 begonnen*
Patienten-Identifikations-Liste (PIL)	*HYPRESS_ Patientenidentifikationsliste_final_10-10-2008*	
Fachinformation Hydrocortison	*Fachinformation-Hydrocortison-100-250-2009-07*	
Working-Instruction	*HYPRESS WI Final 1.2_2012_06_20*	*Version 1.1 wurde nicht genutzt*

3.2 Dokumentation und Datenmanagement

Die Dokumentation der medizinischen Studiendaten werden per RDE (Remote Data Entry-Elektronische Dateneingabe) erfasst. Über das elektronische CRF werden die Daten direkt in die Studiendatenbank der Studienzentrale übernommen.

Den Auditoren werden die Zugangsdaten für das eCRF durch das ZKS Leipzig -KKS zur Verfügung gestellt. Eine entsprechende Schulung zum Umgang mit dem eCRF erfolgt durch den Projektmanager und/oder den Datenmanager der Hypress-Studie.

3.3 Patientenaufklärung und –einwilligung

Die Screening-Untersuchung erfolgt im Rahmen des ITS–Aufenthaltes. Vor Einschluss in die Studie wird der Patient bzw. bei nichteinwilligungsfähigen Patienten sein Betreuer (wenn vorhanden) bzw. der benannte Bevollmächtigte über die Studie aufgeklärt und erklärt die Einwilligung schriftlich. Hierfür existieren separate Dokumente. Ein Exemplar der unterschriebenen Patienten bzw. Betreuer-Einwilligung/Bevollmächtigten-Einwilligung wird im Prüfarztordner abgelegt, das zweite unterschriebene Exemplar oder eine Kopie verbleibt beim Patienten/Betreuer.

Ein nichteinwilligungsfähiger Patient wird, wenn weder Betreuer noch Bevollmächtigter vorhanden sind, über einen unabhängigen Konsiliararzt vorläufig in die Studie eingeschlossen und randomisiert. Eine entsprechende Empfehlung des Konsiliararztes zur Studienteilnahme des Patienten wird im Prüfarztordner abgelegt. Bei Anwendung des Konsiliararztverfahrens muss der Antrag auf Bestellung eines gesetzlichen Betreuers beim zuständigen Vormundschaftsgericht in jedem Fall unverzüglich erfolgen, da nur dieser eine rechtswirksame Einwilligung zur Teilnahme an der Studie geben kann. Im Rahmen des Audits wird die Beantragung einer Betreuung innerhalb von 3 Tagen nach Randomisation des Patienten akzeptiert. Eine nachträgliche Aufklärung des Patienten muss erfolgen, sobald der Zustand des Patienten dies erlaubt. Der Patient muss schriftlich seine Einwilligung zur weiteren Studienteilnahme erteilen.

3.4 Behandlung von vorzeitigen Studienabbrüchen

Beendet ein Patient die Therapie oder die gesamte Studienteilnahme auf eigenen Wunsch oder Wunsch des Betreuers / Bevollmächtigten, ist dies in der Patientenakte und im eCRF (Änderung Studienstatus IE_01 / Rücknahme Einwilligung – REW) entsprechend zu dokumentieren.

Wird nur die Einwilligung zur Studientherapie zurückgezogen, so werden die Patienten, wie im Protokoll vorgesehen, weiter beobachtet und dokumentiert.

Wird die Therapie/Studie abgebrochen, muss dies auch in der Patientenakte dokumentiert werden.

3.5 Patientennummer – Patienten-ID

Durch die internetbasierte Randomisation werden dem Patienten eine Medkit-ID und eine 5-stellige Patienten-ID zugewiesen, der Prüfer erhält die Mitteilung des Ergebnisses der Randomisation per Fax. Die Center-ID ist 2-stellig.

3.6 Fehlende Werte

Bei elektronischer Dateneingabe muss die entsprechende Begründung in die DB eingegeben werden (force reason bzw. query note). Das bedeutet, dass für einen nicht erhobenen Wert/ gemachte Untersuchung eine Begründung eingetragen werden muss.

3.7 Datenkorrekturen

Grundsätzlich sind alle Korrekturen vom Prüfer oder von autorisierten Personen vorzunehmen und werden mit Datum und Unterschrift (Login in der Datenbank) im Audittrail gespeichert.

Der Monitor darf keine Korrekturen im eCRF vornehmen.

Queries werden elektronisch generiert und sind von autorisierten Personen zu bearbeiten.

4 Informationen zum Monitoring

Die ordnungsgemäße und zeitgerechte Durchführung der verschiedenen Monitoring-Besuche ist im Rahmen des ADAMON-Projektes NICHT Bestandteil des Audits.

5 Auditing

5.1 Umfang des Audits

In der Hypress-Studie werden Audits in den im Anhang gelisteten Prüfzentren durchgeführt. Die zu auditierenden Patienten sind der Patientenliste zu entnehmen. Bei Patienten, die randomisiert wurden, die Therapie aber nicht begonnen haben, sind nur die Einwilligungs- und Datenschutzerklärungen sowie der Grund zum Interventionsende im CRF zu überprüfen und die Ergebnisse im Patienten-CRF zu dokumentieren.

Es ist vorgesehen, dass die ersten Audits von jeweils zwei Auditoren in den Zentren durchgeführt werden, die 7, 11 oder 13 Patienten (insgesamt 5 Zentren) rekrutiert haben.

Es folgen die Zentren, die 17, 24, 58 oder 75 Patienten (insgesamt 4 Zentren) rekrutiert haben.

Damit das Audit vor Ort auf maximal eine Woche zu begrenzen ist, wird bei den Zentren mit 58 und 75 Patienten eine nach Rekrutierungszeitpunkt balancierte Stichprobe von insgesamt 50 Patienten gezogen.

Durch die ADAMON-Projektgruppe wird gemeinsam mit den Auditoren individuell entschieden, das Audit in den Zentren, welche nur 2, 3 oder 4 Patienten rekrutiert haben (insgesamt 3 Zentren), von einem Auditor durchführen zu lassen.

5.2 Vorbereitung der Audits

Die Auditoren erhalten das Audit-Manual zusammen mit den unter 3.1 gelisteten Unterlagen zur Vorbereitung der Audits. Anschließend erfolgt eine Vorbesprechung der Audits mit den ADAMON-Projektmitarbeitern. Noch offene Fragen werden durch die ADAMON-Projektmitarbeiter ggf. mit dem Projektmanagement bzw. der Studienleitung der Hypress-Studie geklärt.

In jedem Prüfzentrum übernimmt jeweils ein Auditor die koordinierende Funktion in Bezug auf Planung und Durchführung des Audits sowie der Berichterstattung. Dieser ist erster Ansprechpartner für die Prüfzentren, vereinbart die Termine, führt das Abschlussgespräch und hat die Verantwortung für die Audit-CRFs und Auditberichte.

Der Audittermin wird dem zu auditierenden Prüfzentrum rechtzeitig vor dem geplanten Termin durch die Auditoren schriftlich mitgeteilt. Mit diesem Schreiben werden die Prüfzentren über das Auditziel, den Auditablauf, die Zeitplanung sowie die vom Prüfzentrum bereitzustellenden Auditunterlagen und die Namen der Auditoren informiert. Beim Vorliegen elektronischer Patientenakten ist zu beachten, dass entweder ein separater Zugang für die Auditoren vom Prüfzentrum einzurichten ist oder die Akteneinsicht in Anwesenheit eines Mitarbeiters des Prüfzentrums erfolgen muss.

Voraussetzung für die Audits ist, dass der (Haupt-) Prüfer und die für wesentliche Aufgaben der Studiendurchführung autorisierten Mitarbeiter der Studiengruppe bei den Audits anwesend sind. Mögliche Abweichungen hiervon sind im Auditbericht zu dokumentieren.

5.3 Prüfarztordner (Investigator Site File, ISF)

Eine Überprüfung des ISF erfolgt nur in Bezug auf diejenigen Dokumente, die für die Feststellung von schwerwiegenden oder kritischen Verstößen gegen die Ziele der Good Clinical Practice relevant sind:

- Schutz der Sicherheit, Rechte, Integrität sowie der Vertraulichkeit der Identität der Prüfungsteilnehmer
- Glaubwürdigkeit und Korrektheit der Daten und Ergebnisse.

Im Einzelnen sind dies folgende Dokumente:

- Patientenidentifikationsliste (PIL) ISF Punkt 4

  → Überprüfung, ob von allen Patienten auf der PIL eine unterschriebene Einwilligungserklärung/Datenschutzerklärung der Patienten/Betreuer/Bevollmächtigten *oder* Erklärung Konsiliararzt existiert und umgekehrt

  und Dokumente zum Nachweis der Einrichtung der Eilbetreuung, Kopien der Betreuungsurkunde/Betreuerausweis oder der Vorsorgevollmacht vorliegen

- Personal- und Delegierungsliste (PDL) – ISF Punkt 7

  Die PDL wird im Hinblick auf die vorhandenen Personalressourcen zur Gesamteinschätzung des Prüfzentrums überprüft. Dies bezieht sich im Wesentlichen auf die Anzahl der Prüfer/ärztlichen Mitglieder der Prüfgruppe, Studienassistenten/nicht-ärztlichen Mitglieder der Prüfgruppe, die aktiv an der Studiendurchführung beteiligt waren. Die Überprüfung der Qualifizierung von Prüfern/ärztlichen Mitglieder ist nicht Gegenstand des Audits.

  Vor 2. AMG-Änderungsgesetz

  Prüfer sind Ärzte, für die eine positive Bewertung durch die Ethikkommission vorliegt. Eine entsprechende Auflistung der Prüfer wird zum Audit für jedes Prüfzentrum zur Verfügung gestellt, hierbei ist das Datum der Meldung an die lokale Behörde beim Audit zu berücksichtigen. Die Aufklärung der Patienten/Betreuer/Bevollmächtigten sowie Einholung der Einwilligungserklärungen und Meldung von SAEs müssen durch einen Prüfer erfolgen.

  Sollte die Autorisierung des Prüfers durch den Hauptprüfer auf der PDL fehlen, ist dies im Rahmen des Audits als geringfügiger Mangel zu bewerten.

<u>Nach 2. AMG-Änderungsgesetz</u>

Für die Hypress-Studie wurde durch den Sponsor entschieden, die neue Prüferdefinition nur dann umzusetzen, wenn ein Wechsel des ärztlichen Personals im Prüfzentrum erfolgte. Für das Audit wird eine Auflistung der Prüfer/Stellvetreter/ärztlichen Mitarbeiter für jedes Prüfzentrum zur Verfügung gestellt. Ärztliche Mitarbeiter sind durch den Prüfer zu benennen.

Hinsichtlich ihrer Qualifikation werden Erfahrungen in klinischen Studien vorausgesetzt bzw. die Teilnahme an einem GCP Kurs durch den Sponsor empfohlen.

Anhand der PDL sind Aufgaben an die Mitglieder der Prüfgruppe durch den Prüfer delegiert worden. Dies ist beim Audit nur bei den Aufgaben 1=Aufklärung der Patienten u. Einholung der Einwilligungserklärung sowie 2=Meldung von SAEs zu berücksichtigen.

Sollte die Autorisierung des Prüfers hinsichtlich der o.g. Aufgaben 1 und 2 auf der PDL fehlen, ist dies im Rahmen des Audits als schwerwiegender/kritischer Mangel zu bewerten. Eine Überprüfung der Qualifikation ärztlicher Mitarbeiter ist nicht Bestandteil des Audits.

- Primär- und Follow-up Meldungen schwerwiegender unerwünschter Ereignisse - ISF Punkt 14

  Bei den SAE-Meldungen sind die Angaben zu Patient-ID, Event (Event, Date and time of onset, Causal relationship, Serious event) zu überprüfen. Weitere Angaben zum Event (Intensity, Outcome) sowie IMP, Medical history / Concomitant therapy, Lab findings werden nicht auditiert.

Die oben genannten Dokumente stellen die Referenzdokumente dar, auf welche sich das Audit des ISF bezieht. Diese essentiellen Studiendokumente sind hinsichtlich ihrer vollständigen Ablage im ISF zu kontrollieren.

5.4 Einschätzung des Prüfzentrums

Jedes Prüfzentrum wird im Rahmen der Audits von den Auditoren beurteilt. Hierzu sind die Personalressourcen bezogen auf die PDL (siehe Punkt 5.3), die Organisationsstruktur des Prüfzentrums sowie das prüfplankonforme und GCP-konforme Arbeiten des Studienpersonals im Rahmen der Hypress Studie im Allgemeinen Audit CRF zu beschreiben, soweit diese Kriterien beim Audit einschätzbar sind. Eine zusammenfassende Einschätzung des Prüfzentrums wird durch den Auditbericht an den Sponsorbevollmächtigten kommuniziert.

5.5 Quelldokumente

In jedem Prüfzentrum wird überprüft, welche Arten von Quelldokumenten für die Patienten vorhanden sind und auf welche Arten von Quelldokumenten der Auditor Zugriff hat. Die entsprechenden Informationen werden schriftlich im patientenbezogenen Audit CRF festgehalten.

Es gibt keine im Prüfplan vordefinierten Direkteinträge. Sollten Arztbriefe als Quelldokumente existieren, die nicht im Original unterschrieben vorliegen, dient die jeweils letzte abgelegte Version dieser Briefe als Quelldokument.

Wenn zum Audit im Prüfzentrum Arztbriefe der einzelnen Patienten vorliegen, die umfassende Informationen hinsichtlich aufgetretener AEs/SAEs enthalten, können diese Arztbriefe als Quelldokument herangezogen werden.

Hinweis: Für das Audit werden zwingend vollständige Quelldaten für den Aufenthalt auf ITS erwartet. Wurde der Patient vor Tag 14 von der ITS entlassen, wird nur die Zeit auf ITS betrachtet <u>oder</u> wird nur dann der gesamte Zeitraum bis Tag 14 bzw. Tag 28 betrachtet, wenn den Auditoren hierfür neben den ITS-Akten die weiteren Quelldaten zur Verfügung stehen.

Dies gilt für die Überprüfung der prüfplangemäßen Therapie, des primären Endpunktes und der ausgewählten sekundären Endpunkte sowie der Meldung von SAEs.

5.6 Verstöße gegen die Ziele der GCP

Das primäre Zielkriterium des ADAMON-Projektes ist das Auftreten mindestens eines **kritischen oder schwerwiegenden Verstoßes gegen GCP**, das beim Audit festgestellt wird.

Einstufung der Mängel

**Kritische Mängel**	
Definition	Gegebenheiten, Methoden oder Prozesse, die die **Rechte**, die **Sicherheit** oder das **Wohlergehen** der Probanden/Patienten und/oder die **Qualität und Integrität der Daten negativ beeinflussen**.<br>Kritische Mängel werden als nicht akzeptabel angesehen.
Mögliche Konsequenzen	Ablehnung der Daten und/oder Einleitung rechtlicher Schritte
Bemerkungen	Üblicherweise gilt: Als kritisch klassifizierte Mängel können sich aus einem Muster von schwerwiegenden Mängeln, schlechter Datenqualität und/oder dem Fehlen von Quelldaten ergeben. Dies wird im Rahmen der ADAMON-Audits nicht berücksichtigt, da hier eine Beurteilung von Mängeln bei jedem einzelnen Studienteilnehmer erfolgt. Eine Gesamtbeurteilung des Zentrums ist nicht Ziel der ADAMON-Audits.<br>Betrug gehört ebenfalls zu den kritischen Mängeln

**Schwerwiegende Mängel**	
Definition	Gegebenheiten, Praktiken/Methoden oder Prozesse, die die Rechte, die Sicherheit oder das Wohlergehen der Probanden/Patienten und/oder die Qualität und Integrität der Daten **negativ beeinflussen könnten**.<br>Schwerwiegende Mängel sind erhebliche Defizite und direkte Verstöße gegen GCP Prinzipien.
Mögliche Konsequenzen	Mögliche Ablehnung der Daten und/oder Einleitung rechtlicher Schritte
Bemerkungen	Üblicherweise gilt: Als schwerwiegend eingestufte Mängel können sich aus einem Muster von Abweichungen und/oder mehreren geringfügigen Mängeln ergeben. Dies wird im Rahmen der ADAMON-Audits nicht berücksichtigt, da hier eine Beurteilung von Mängeln bei jedem einzelnen Studienteilnehmer erfolgt. Eine Gesamtbeurteilung des Zentrums ist nicht Ziel der ADAMON-Audits.

**Geringfügige Mängel**	
Definition	Gegebenheiten, Praktiken/Methoden oder Prozesse von denen **nicht erwartet wird, dass sie einen negativen Einfluss** auf die Rechte, die Sicherheit oder das Wohlergehen der Probanden/Patienten und/oder die Qualität und Integrität der Daten **haben**.
Mögliche Konsequenzen	Geringfügige Mängel sind Hinweise für die Notwendigkeit, die Gegebenheiten, Methoden oder Prozesse zu verbessern.
Bemerkungen	Üblicherweise gilt: Viele geringfügige Mängel können ein Indiz für eine insgesamt schlechte Qualität sein und zusammengenommen mit einem schwerwiegenden Mangel und dessen Konsequenzen gleichgestellt werden. Dies wird im Rahmen der ADAMON-Audits nicht berücksichtigt, da hier eine Beurteilung von Mängeln bei jedem einzelnen Studienteilnehmer erfolgt. Eine Gesamtbeurteilung des Zentrums ist nicht Ziel der ADAMON-Audits.

**Kommentar**	
Definition	Die Feststellungen können zu Vorschlägen führen, wie die Qualität verbessert oder das Fehlerpotenzial in Zukunft verringert werden kann.

Im Folgenden werden mögliche patientenbezogene Mängel definiert und ihr Schweregrad eingeschätzt, wobei kritische und schwerwiegende Mängel zu einer Kategorie zusammengefasst werden. **Gegenstand des Audits sind nur Patienten, die auch zur Studienpopulation gehören. Diese sind der Patientenliste zu entnehmen.**

5.6.1 Mängel bei Aufklärungsprozess und Einwilligungserklärung/Datenschutzerklärung

Einwilligungsfähiger Patient

Die Screening-Untersuchung erfolgt im Rahmen des ITS–Aufenthaltes. Vor Einschluss in die Studie wird der Patient über die Studie aufgeklärt und erklärt seine Einwilligung schriftlich.

→ HYPRESS_Information-Einwilligung_Patient_v2.0_2008-08-19-final
Wenn ein Patient zum Zeitpunkt der Aufklärung ansprechbar aber nicht schreibfähig ist, kann die mündliche Einwilligung des Patienten durch einen Zeugen (Person, die nicht zum Studienpersonal gehört) schriftlich bestätigt werden.

→ HYPRESS_Information-Einwilligung_Patient_v2.0_2008-08-19-final

Nichteinwilligungsfähiger Patient

Bei nichteinwilligungsfähigen Patienten kann der Betreuer (wenn vorhanden) bzw. der benannte Bevollmächtigte über die Studie aufgeklärt werden und seine Einwilligung zur Studienteilnahme des Patienten schriftlich erklären.

→ HYPRESS_Information-Einwilligung_Betreuer_v2.0_2008-08-19-final

→ HYPRESS_Information-Einwilligung_Bevollmächtigter_v2.0_2008-08-19-final

Falls der Patient weder einen Betreuer noch Bevollmächtigten hat, kann er vorläufig in die Studie eingeschlossen werden. Hierfür ist vorab eine Bestätigung der Nichteinwilligungsfähigkeit des Patienten und eines möglichen Nutzens durch die Studientherapie durch einen unabhängigen Konsiliararzt notwendig, die im ISF abgelegt wird. Bei Anwendung des Konsiliararztverfahrens muss der Antrag auf Bestellung eines gesetzlichen Betreuers unverzüglich erfolgen, da nur dieser eine rechtswirksame Einwilligung zur Teilnahme an der Studie geben kann. Im Rahmen des Audits wird die Beantragung einer Betreuung innerhalb von 3 Tagen nach Randomisation des Patienten akzeptiert. Erfolgt der Einschluss des Patienten nach Bestätigung eines Konsiliararztes, muss sobald möglich, eine schriftliche Einwilligung durch den Patienten/ Betreuer eingeholt werden.

→ 07d_HYPRESS_Erklaerung Konsiliar_v2.0_2008-08-19_final

Sobald ein Betreuungsverhältnis eingerichtet wurde, ist der Betreuer über die Studie aufzuklären und muss seine Einwilligung zur weiteren Studienteilnahme des Patienten durch Datum und Unterschrift bestätigen.

→ HYPRESS_Information-Einwilligung_Betreuer_v2.0_2008-08-19-final

Sollte der Patient vor Einrichtung des Betreuungsverhältnisse wieder einwilligungsfähig sein, so ist er über die Studie aufzuklären und muss seine Einwilligung zur weiteren Studienteilnahme durch Datum und Unterschrift bestätigen.

→ HYPRESS_Information-Einwilligung_Patient_v2.0_2008-08-19-final

Hinweise: Vor Studieneinschluss sind der mutmaßliche Patientenwille und das Vorliegen einer Patientenverfügung zu beachten. Hierzu ist es erforderlich, die Angehörigen von nichteinwilligungsfähigen Patienten zu befragen und ggf. Kontakt zu den Angehörigen aufzunehmen, falls diese zum Zeitpunkt des Studieneinschlusses nicht anwesend sind.

Sollte zum Zeitpunkt der Aufklärung/Einwilligung bekannt sein, dass eine Vorsorgevollmacht oder ein Betreuungsverhältnis für den nichteinwilligungsfähigen Patienten vorhanden ist, muss dies unbedingt berücksichtigt werden. Auch hier sollten die Angehörigen umgehend kontaktiert und befragt werden.

Anhand der Patientenakte muss nachvollziehbar sein, dass die Angehörigen zu den o.g. Punkten befragt wurden. **Ein systematisches Nichtkontaktieren der Angehörigen ist als kritischer oder schwerwiegender Mangel zu bewerten und im allgemeinen CRF zu dokumentieren.**

Für den Fall, dass erst nach Einschluss in die Studie die o.g. Informationen bekannt wurden, ist dies bei der Einschätzung durch die Auditoren entsprechend zu berücksichtigen und nachvollziehbar zu dokumentieren.

Nachträgliche Aufklärung und EWE des Patienten
Wenn ein Patient zum Zeitpunkt des Screenings nicht einwilligungsfähig ist, muss sobald möglich und zumutbar, eine Einwilligung des Patienten zur weiteren Teilnahme erfolgen.

Beim Audit ist anhand der Quelldokumentation zu überprüfen, ob der Patient im Studienverlauf wieder einwilligungsfähig wurde und seine Einwilligung zur weiteren Studienteilnahme durch Unterschrift vorliegt.

Eine Überprüfung der nachträglichen Unterschrift des Patienten auf der Datenschutzerklärung ist im Rahmen des Audits nur dann erforderlich, falls die Datenschutzerklärung primär nicht vom Betreuer/Bevollmächtigten unterschrieben wurde.

→ HYPRESS_Information-Einwilligung_Patient_v2.0_2008-08-19-final

In der HYPRESS-Studie sind folgende Besonderheiten zu beachten:

Nach Rücksprache mit der ff EK dürfen die Daten von Patienten, die innerhalb von 3 Tagen nach Randomisation versterben (bevor EWE eingeholt bzw. ein Betreuer bestellt werden konnte) komplett für die Auswertung verwendet werden. Die Daten dieser Patienten wurden nicht monitoriert und dürfen auch nicht auditiert werden, da keine Datenschutzerklärung vorliegt.

Falls der bestellte Betreuer der Studienteilnahme eines Patienten nicht zustimmt, dürfen die bis zu diesem Zeitpunkt erhobenen Daten für Safety-Analysen verwendet aber nicht auditiert werden, da keine Datenschutzerklärung vorliegt.

Wenn ein wieder einwilligungsfähiger Patient die EWE zurücknimmt, nachdem ein Betreuer/ Bevollmächtigter eingewilligt sowie die Datenschutzerklärung unterschrieben hat, dürfen die Daten bis zum Zeitpunkt der Rücknahme auditiert werden.

Zu beachten ist, dass die Daten von Patienten, bei denen keine Datenschutzerklärung vorliegt, nicht auditiert werden dürfen. Die Datenschutzerklärung kann nur von Patient, Betreuer oder Bevollmächtigten unterschrieben werden.

Weiterhin ist auf dem Formular der Einwilligungserklärung die Dokumentation des Ortes der Aufklärung bzw. Einwilligung vorgesehen. Da die Ortsangabe laut GCP nicht gefordert wird, ist diese beim Audit auch nicht zu berücksichtigen.

Die wissenschaftlichen Begleitprojekte – Fragebogen zur Posttraumatischen Belastungsreaktion und krankheitsbezogene Lebensqualität, Immunmonitoring, ACTH-Test sowie DNA-Probe und die damit verbundene Datenerhebung – sind nicht Bestandteil des Audits.

**EWE bei primär einwilligungsfähigen Patienten**	**kritisch oder schwerwiegend**	**geringfügig**
EWE vom Patienten liegt nicht vor	X	
EWE ist dem Patienten weder durch Namen noch durch Patienten-Nr. zuzuordnen.	X	
EWE liegt nur in Kopie vor.		X
Mängel hinsichtlich Einwilligung durch Patient		
Die Unterschrift des Patienten fehlt	X	
Das eigenhändige Datum des Patienten fehlt<br>und es lässt sich nicht anhand eines zeitgleichen Eintrags in der Patientenakte/ NTF nachvollziehen, dass die Einwilligung vor Randomisation erfolgt ist.<br>Hinweis: falls anhand Akte/NTF nachvollziehbar oder Datum nachträglich durch Unterschrift des Patienten schriftlich bestätigt -> geringfügiger Mangel	X	
Das Datum des Patienten ist angegeben, wurde aber nicht vom Patienten eingetragen,<br>jedoch wurde das Einwilligungsdatum nachträglich durch Unterschrift des Patienten schriftlich bestätigt.		X
Das eigenhändige Datum des Patienten liegt nach Randomisation<br>und es lässt sich nicht anhand eines zeitgleichen Eintrags in der Patientenakte nachvollziehen, dass die Einwilligung vor Randomisation erfolgt ist.<br>Hinweis: falls anhand Akte nachvollziehbar -> geringfügiger Mangel	X	
Mängel hinsichtlich Bestätigung der mündlichen Einwilligung durch unabhängigen Zeugen		

**EWE bei <u>primär einwilligungsfähigen Patienten</u>**	**kritisch oder schwerwiegend**	**geringfügig**
Unterschrift stammt von einem Zeugen, der zum Prüfteam (siehe PDL) oder Stationspersonal gehört	X	
Unterschrift des unabhängigen Zeugen fehlt	X	
Unabhängiger Zeuge hat EWE nicht eigenhändig datiert<br><u>und</u> es lässt sich nicht anhand eines zeitgleichen Eintrags in der Patientenakte/ NTF nachvollziehen, dass die mündliche Einwilligung des Patienten vor Randomisation erfolgt ist.<br><u>Hinweis:</u> falls anhand Akte/ NTF nachvollziehbar -> geringfügiger Mangel	X	
Mängel hinsichtlich Aufklärung durch Prüfer/qualifizierten ärztlichen Mitarbeiter		
Die Unterschrift des aufklärenden Prüfers/qualifizierten ärztlichen Mitglieds der Prüfgruppe fehlt.	X	
<u>Vor 2. AMG-Änderungsgesetz</u><br>Aufklärung/Unterschrift erfolgte durch einen Arzt, für den eine zustimmende Bewertung durch die EK vorliegt, der <u>aber</u> nicht durch den Hauptprüfer auf der Personal- und Delegierungsliste (PDL) autorisiert wurde.		X
<u>Vor 2. AMG-Änderungsgesetz</u><br>Die Aufklärung/Unterschrift erfolgte durch einen Arzt, für welchen jedoch keine zustimmende Bewertung durch die EK vorliegt.<br>Hinweis: es spielt hier keine Rolle, ob der Arzt lt. PDL autorisiert war oder nicht	X	
<u>Nach 2. AMG-Änderungsgesetz</u><br>Die Aufklärung/Unterschrift erfolgte durch einen Arzt, der nicht durch den Prüfer auf der PDL hierfür autorisiert wurde.	X	
Das Datum der Unterschrift des aufklärenden Prüfers/qualifizierten ärztlichen Mitglieds der Prüfgruppe fehlt *<u>oder</u>* liegt nach Randomisation<br><u>und</u> es lässt sich nicht anhand des Datums der Einwilligung durch den Patienten nachvollziehen, dass die Aufklärung vor Randomisation stattgefunden hat.<br><u>Hinweis:</u> falls anhand Akte oder Datum der EWE nachvollziehbar -> geringfügiger Mangel	X	
Das Datum der Unterschrift des aufklärenden Prüfers/qualifizierten ärztlichen Mitglieds der Prüfgruppe liegt vor dem Datum der Einwilligung durch den Patienten/ Betreuer/ Bevollmächtigten		X
Sonstiges Problem (Beschreibung notwendig)		

**EWE bei <u>nicht-einwilligungsfähigen</u> Patienten**	**kritisch oder schwerwiegend**	**geringfügig**
Falsche Vorlage zur Information und Einwilligung genutzt<br><u>Hinweis</u>: Es existieren Vorlagen für den Patienten, den Betreuer und den Bevollmächtigten, die inhaltlich identisch sind.		X
Mutmaßlicher Patientenwille wurde nicht beachtet soweit zum Studieneinschluss bekannt	X	
Vorhandensein einer Vorsorgevollmacht wurde nicht berücksichtigt obwohl zum Studieneinschluss bekannt	X	
Vorhandensein eines Betreuungsverhältnisses wurde nicht berücksichtigt obwohl zum Studieneinschluss bekannt	X	
EWE vom Betreuer/Bevollmächtigten liegt nicht vor<br>Hinweis: Nur zutreffend, wenn Betreuer/Bevollmächtigter zum Studieneinschluss vorhanden oder Betreuer später bestellt wurde.	X	
EWE durch vermeintlichen Betreuer/Bevollmächtigten erfolgt, aber Nachweis, wie Kopie der Vorsorgevollmacht oder Betreuungsurkunde, liegt nicht vor	X	

**EWE bei <u>nicht-einwilligungsfähigen</u> Patienten**	**kritisch oder schwerwiegend**	**geringfügig**
EWE vom Betreuer/Bevollmächtigten ist Patient weder durch Namen noch durch Patienten-Nr. zuzuordnen.	X	
EWE vom Betreuer/Bevollmächtigten liegt nur in Kopie vor.		X
Mängel hinsichtlich Einwilligung durch Betreuer/Bevollmächtigten		
Die Unterschrift des Betreuers/Bevollmächtigten fehlt	X	
Das eigenhändige Datum des Bevollmächtigten fehlt,<br><u>und</u> es lässt sich nicht anhand eines zeitgleichen Eintrags in der Patientenakte/NTF nachvollziehen, dass die Einwilligung des Bevollmächtigten vor Randomisation erfolgt ist.<br><u>Hinweis:</u> falls anhand Akte/NTF nachvollziehbar oder Datum nachträglich durch Unterschrift des Bevollmächtigten schriftlich bestätigt -> geringfügiger Mangel	X	
Das eigenhändige Datum des Betreuers fehlt,<br><u>und</u> es lässt sich nicht anhand eines zeitgleichen Eintrags in der Patientenakte/NTF nachvollziehen, dass die Einwilligung nach Einrichtung des Betreuungsverhältnisses erfolgt ist.<br><u>Hinweis:</u> falls anhand Akte/NTF nachvollziehbar oder Datum nachträglich durch Unterschrift des Betreuers schriftlich bestätigt -> geringfügiger Mangel	X	
Das Datum des Betreuers/Bevollmächtigten ist angegeben, wurde aber nicht vom Betreuer/Bevollmächtigten eingetragen,<br><u>jedoch</u> wurde das Einwilligungsdatum nachträglich durch Unterschrift des Betreuers/ Bevollmächtigten schriftlich bestätigt.		X
Es existiert bereits vor Einschluss ein Betreuungsverhältnis bzw. es liegt eine Vorsorgevollmacht vor, das eigenhändige Datum des Betreuers/ Bevollmächtigten liegt trotzdem nach Randomisation<br><u>und</u> es lässt sich nicht anhand eines zeitgleichen Eintrags in der Patientenakte nachvollziehen, dass die Einwilligung vor Randomisation erfolgt ist.<br><u>Hinweis:</u> falls anhand Akte nachvollziehbar -> geringfügiger Mangel	X	
Mängel hinsichtlich Einwilligung durch Patient		
Falls zutreffend: Nachträgliche Unterschrift des Patienten fehlt	X	
Mängel hinsichtlich Erklärung Konsiliararzt, falls zutreffend		
Konsiliararzt nicht unabhängig von Prüfstelle<br><u>Hinweis</u>: Der Konsiliararzt darf nicht an der Studie beteiligt sein, nicht zum Betreuungsteam des Patienten gehören und auch nicht der studiendurchführenden Station angehören. Die entsprechende Überprüfung erfolgt in der Regel anhand des Stempels auf der Konsiliararzterklärung.	X	
Erklärung des Konsiliararztes über die Studienteilnahme liegt nicht vor	X	
Konsiliararzterklärung ist dem Patienten weder durch Namen noch durch Patienten-Nr. zuzuordnen.	X	
Konsiliararzt hat die Studienteilnahme nicht befürwortet, Patient wurde trotzdem in die Studie eingeschlossen	X	
Datum der Konsiliarzterklärung liegt nach Randomisierung	X	
Betreuungsverhältnis wurde nicht innerhalb von 3 Kalendertagen nach dem Tag der Randomisation beantragt und Patient ist nicht innerhalb von 3 Kalendertagen wieder einwilligungsfähig.<br><u>Hinweis</u>: In der Akte muss die Beantragung belegt sein, z.B. Fax an Amtsgericht.	X	
Sonstiges Problem (Beschreibung notwendig)		

**Datenschutzerklärung**<br>*Bei Patienten, die primär nicht einwilligungsfähig waren, ist eine Überprüfung der nachträglichen Unterschrift des Patienten auf der Datenschutzerklärung im Rahmen des Audits nur dann erforderlich, falls die Datenschutzerklärung nicht vom Betreuer/Bevollmächtigten unterschrieben wurde.*	**kritisch oder schwerwiegend**	**geringfügig**
DSE liegt nicht vor.	X	
DSE ist dem Patienten nicht zuzuordnen weder durch Namen noch durch Patienten-Nr. oder als Anhang zur EWE.	X	
DSE liegt nur in Kopie vor.		X
Mängel hinsichtlich Unterschrift durch Patient/Betreuer/Bevollmächtigten		
Die Unterschrift des Patienten/Betreuers/Bevollmächtigten fehlt.	X	
Das Datum der Unterschrift zum Datenschutz des Patienten/Betreuers/Bevollmächtigten fehlt.		X
Das Datum der Unterschrift zum Datenschutz des Patienten/Betreuers/Bevollmächtigten ist angegeben, wurde aber nicht vom Patienten/ Betreuer/Bevollmächtigten eingetragen.<br>Hinweis: Weitere Mängel beim Datum sind auch als geringfügig zu bewerten.		X
Sonstiges Problem (Beschreibung notwendig)		

5.6.2 Mängel bei Ein- und Ausschlusskriterien

Betrachtet wird hier die Missachtung sicherheitsrelevanter oder wirksamkeitsrelevanter Ein- und Ausschlusskriterien. Voraussetzung für den Einschluss der Patienten ist das Vorliegen einer schweren Sepsis, deren Beginn nicht länger als 48h zurückliegen darf.

Im Folgenden werden nur diejenigen Ein- und Ausschlusskriterien aufgeführt, die im Rahmen des ADAMON Audits zu überprüfen sind. Die Nummer entspricht dabei der Nummer des Kriteriums im Prüfplan.

Bei der folgenden Auflistung ist zu beachten, dass ausgewählte Ausschlusskriterien beim ADAMON Audit nur zu berücksichtigen sind, wenn explizit Hinweise auf deren Existenz in der Patientenakte zu finden sind.

**Verletzung der Einschlusskriterien**	**kritisch oder schwerwiegend**	**geringfügig**
**III. Kein klinischer Nachweis einer *INFEKTION***<br>***d.h. keines der unten aufgeführten Kriterien trifft zu***<br>Hinweis: Mindestens **1** Kriterium zum Nachweis einer Infektion erforderlich:<br>*Kriterien nach Häufigkeit des Auftretens sortiert*<br>**d.** Klinisch vermutete Infektion ohne Nachweis pathogener Mikroorganismen (z.B. neues pulmonales Infiltrat im Rö-Thorax, behandelte Pneumonie, Purpura fulminans, nekrotisierende Fasciitis)<br>**b.** Identifizierbarer Fokus (z.B. purulentes Sputum oder Wundsekret, Darmperforation)<br>**a.** Nachweis pathologischer Mikroorganismen in Blut, Sputum, Urin oder normalerweise sterilem Körpergewebe<br>**c.** Nachweis von Granulozyten in normalerweise sterilem Gewebe	X	
**IV. Kein Nachweis von *SIRS* -** Systemisches inflammatorisches Response-Syndrom<br>***d.h. nur maximal eines der unten aufgeführten Kriterien trifft zu***<br>Hinweis: Mindestens **2** Kriterien zum Nachweis von SIRS erforderlich:<br>*Kriterien nach Häufigkeit des Auftretens sortiert*<br>**b.** Tachykardie (≥ 90/min)	X	

Verletzung der Einschlusskriterien	kritisch oder schwerwiegend	geringfügig
**c.** Tachypnoe (≥ 20/min) oder Hyperventilation ($PaCO_2$ ≤ 32mmHg, ≤ 4,4 kPa) oder maschinelle Beatmung<br>**a.** Fieber (≥38 °C) oder Hypothermie (≤ 36 °C)<br>**d.** Leukozytose (≥ 12,000/µl) oder Leukopenie (≤ 4,000/µl) oder ≥ 10% unreife Granulozyten		
**V. Kein Nachweis der *ORGANDYSFUNKTION***<br>***d.h. keines der unten aufgeführten Kriterien trifft zu***<br>Hinweis: Mindestens **1** Kriterium zum Nachweis der Organdysfunktion erforderlich:<br>*Kriterien nach Häufigkeit des Auftretens sortiert*<br>**d. Pulmonale Dysfunktion/Hypoxämie:** PaO_2 ≤ 75 mmHg (≤ 10 kPa) bei Raumluft oder PaO_2/FiO_2 ≤ 250 mmHg (≤ 33 kPa) unter O_2-Applikation. Die Hypoxämie darf nicht durch kardiale oder pulmonale Ursache bedingt sein (z.B. Emphysem).<br>**b. Akute renale Dysfunktion:** Urinausscheidung Oligurie ≤ 0,5 ml/kg/Std. über mindestens 2 Stunden trotz ausreichender Volumensubstitution und/oder Kreatinin-Anstieg > 2 x des oberen Normalwertes und/oder Nierenersatztherapie wegen Sepsis-induziertem Nierenversagen<br>**e. Mirkrozirkulatorische Dysfunktion:** Laktat > 1,5 x des oberen Normalwertes und/oder Basendefizit ≥ 5 mmol/l und/oder metabolische Azidose mit pH < 7,3 und /oder gestörte kapillare Reperfusion und/oder Marmorierung der Haut und/oder signifikante Ödeme bei Kapillarlecksyndrom.<br>**a. Enzephalopathie** ohne Beeinflussung durch psychotrope Pharmaka: Vigilanzstörung, Unruhe, Delirium, Desorientiertheit<br>**c. Koagulopathie:** Thrombozyten ≤ 100,000/µl oder > 30 % Abfall innerhalb von 24 Stunden; Die Thrombozytopenie darf nicht hämorrhagisch oder immunologisch bedingt sein.	X	
***ORGANDYSFUNKTION* ≥ 48 Stunden**	X	
Sonstiges Problem (Beschreibung notwendig)		

Verletzung der Ausschlusskriterien	kritisch oder schwerwiegend	geringfügig
**I. Sepsis-induzierte *HYPOTENSION*** trotz adäquatem Volumenstatus, wenn eines der Kriterien für mindestens 4 h erfüllt ist:<br>- mittlerer arterieller Druck (MAD) < 65 mmHg (< 8,7 kPa) ***oder***<br>- systolischer arterieller Druck (SAD) < 90 mmHg (< 12 kPa) ***oder***<br>- der Einsatz von Vasopressoren zur Aufrechterhaltung:<br>• **des SAD ≥ 90 mmHg (≥ 12 kPa)** oder<br>• des MAD ≥ 65 mmHg (≥ 8,7 kPa)<br>Der adäquate Volumenstatus ist definiert als: zentravenöser Druck (ZVD) ≥ 8 mmHg ohne maschinelle Beatmung oder ZVD ≥ 12 mmHg mit maschineller Beatmung **und** zentralvenöse Sauerstoffsättigung ($ScvO_2$) ≥ 70 %.<br>Zu den Vasopressoren gehören: Dopamin ≥ 5 µg/kg/min oder jede Dosis von Adrenalin, Noradrenalin, Vasopressin oder andere Vasopressoren.<br>Patienten, die nur vorübergehend Vasopressoren (z.B. initiale Volumengabe, Intubation, Anästhesie) erhalten, befinden sich nicht im septischen Schock, wenn sie danach für mindestens 2 Stunden vasopressorfrei und nicht hypotensiv sind und können in die Studie eingeschlossen werden.	X	

Verletzung der Ausschlusskriterien	kritisch oder schwerwiegend	geringfügig
**III.** Patienten mit Glukokortikoid-Medikation und bei denen eine Fortsetzung der Medikation bzw. eine Substitutionstherapie indiziert ist (z.B. > 10 mg Prednisolon-Äquivalent pro Tag für mindestens 5 Tage innerhalb der letzten 3 Monate)<br>Hinweis: Eine Therapie mit topischen und/oder inhalativen Glukokortikoiden ist kein Ausschlusskriterium, wenn keine Indikation für eine Weiterbehandlung mit systemischen Glucocorticoiden besteht.	X	
**IX.** Alter < 18 Jahre	X	
**Verletzung der Ausschlusskriterien – nur zu berücksichtigen, falls entsprechende Hinweise in der Akte zu finden sind**	**kritisch oder schwerwiegend**	**geringfügig**
**II.** Hinweis auf Unverträglichkeit gegen Hydrocortison-21-hydrogensuccinat, Natriummonohydrogenphosphat oder Mannitol (Placebo).	X	
**IV.** Hinweis auf andere Indikation für systemische Glukokortikoid-Therapie (z.B. Anaphylaxie, COPD, Asthma, Autoimmunerkrankungen)	X	
**V.** Hinweis auf Therapielimitierung (DNR-Order)<br>Hinweis: do not resuscitate **-** Die Information ist der Patientenverfügung zu entnehmen, soweit vorhanden.	X	
**VII.** Hinweis auf Schwangerschaft	X	
**X.** Hinweis auf Teilnahme an einer anderen interventionellen klinischen Studie innerhalb der letzten 30 Tage	X	
Sonstiges Problem (Beschreibung notwendig)		

5.6.3 Mängel bei prüfplangemäßer Therapie im Studienverlauf

Bei der internetbasierten Randomisation wurden dem Patienten eine Medkit-ID und eine Patienten-ID zugewiesen, der Prüfer erhielt die Mitteilung des Ergebnisses der Randomisation per Fax. Den Auditoren werden diese Informationen anhand der Patientenlisten zur Verfügung gestellt.

Folgende Applikation und Dosierung des Prüfproduktes (Verum oder Placebo) waren laut Prüfplan vorgegeben - Patienten erhalten maximal 17 Vials:

- 50 mg Initialdosis (Bolus)
- Tag 1[1] bis 5: 200 mg/24h (2x100 mg - 2x 1 Vial)
- Tag 6 bis 7: 100 mg/24h (1x100mg – 1x 1 Vial)
- Tag 8 bis 9: 50 mg/24h (1x 50mg – 1 x ½ Vial)
- Tag 10 und 11: 25 mg/24h (1x25 mg – 1x ¼ Vial)

Beim Audit wird nur die Gabe des Prüfproduktes auf der ITS monitoriert, da bei Verlegung des Patienten auf eine andere Station mit weiterer Gabe des Prüfproduktes der Quelldatenvergleich nicht sichergestellt werden kann.

Beim Audit sind bis zum Tag der ITS Entlassung die Einträge zur Medkit-ID und die Angaben zu den Infusionen (Dosis gesamt) in der Akte mit dem eCRF (CRF Seite Prüfmedikation – IMP) abzugleichen. Sollten beim Audit Abweichungen zwischen CRF und Akte hinsichtlich der dokumentierten einzelnen Infusionen festgestellt werden, dann ist ebenfalls zu überprüfen, ob sich diese Abweichung auf die insgesamt verabreichte Gesamtdosis des Prüfproduktes auswirkt. Eine Übersicht zum Prüfprodukt wird anhand der Patientenliste für das Audit zur Verfügung gestellt.

Wenn die Anzahl der Tage mit Prüfmedikation ohne medizinischen Grund von den Vorgaben im Prüfplan abweicht, ist die verabreichte Gesamtdosis bezogen auf den gesamten Therapiezeitraum bis Studientag 11 bei der Bewertung zu berücksichtigen.

[1] Tag entspricht hier dem 24 Std.-Intervall der Medikationsgabe

<u>Hinweis:</u> Zum Zeitpunkt der Gabe des Prüfproduktes durfte kein septischer Schock bestehen, da dies dem primären Endpunkt entsprechen würde. Entwickelte der Patient einen septischen Schock NACH Randomisation und VOR GABE des Prüfproduktes, war die Studie abzubrechen. Die Therapie war abzubrechen, wenn der septische Schock nach Beginn der Applikation des Prüfproduktes aufgetreten war, die Patienten verblieben aber weiterhin in der Studie.

**Prüfplangemäße Therapie**<br>**CRF Seite: IMP, IE_01**	**kritisch oder schwerwiegend**	**geringfügig**
Beginn der ersten Infusion (Bolus) erfolgte vor Randomisierung oder ≥ 4h nach Randomisation (Ausschluss: septischer Schock)	X	
Therapiebeginn obwohl septischer Schock bestand<br><u>Hinweis:</u> Definition septischer Schock siehe Abschnitt zum primären Endpunkt.	X	
Kein Therapieabbruch im Studienverlauf nachdem Kriterien für septischen Schock für mindestens 4h erfüllt waren	X	
Angabe zur MedKit-ID im CRF nicht übereinstimmend mit den Quelldaten des Patienten (Randofax und/oder Drug Accountability) bzw. nicht anhand der Quelldaten nachvollziehbar	X	
Angaben zur verabreichten Gesamtdosis auf der ITS in Quelldaten und CRF abweichend <u>und</u> mindestens eine der beiden Gesamtdosen (CRF und/oder Quelldaten) weicht um ± 20% von den Vorgaben im Prüfplan ab	X	
Gesamtdosis auf der ITS ohne medizinischen Grund um ± 20% abweichend von den Vorgaben im Prüfplan<br><u>Hinweis:</u> Falls die Angaben zu einzelnen Infusionen zwischen CRF und Quelldaten abweichen, ist auch die Auswirkung auf die insgesamt verabreichte Gesamtdosis des Prüfproduktes auf der ITS zu überprüfen.	X	
Anzahl der Tage mit Prüfmedikation entspricht nicht dem Therapiezeitraum laut Prüfplan <u>und</u> Gesamtdosis um ± 20% abweichend <u>ohne</u> dass medizinische Gründe vorliegen.<br><u>Hinweis:</u> Falls Anzahl der Therapietage abweichend, die Gesamtdosis aber der Vorgabe im Prüfplan entspricht → geringfügiger Mangel	X	
Sonstiges Problem (Beschreibung notwendig)		

5.6.4 Mängel bei Bestimmung des primären Endpunktes

Das Hauptzielkriterium in der HYPRESS-Studie ist das Auftreten eines **septischen Schocks innerhalb von 14 Tagen**. Dieser ist definiert als <u>Sepsis-induzierte HYPOTENSION</u> trotz adäquatem Volumenstatus.

Mindestens <u>eines der folgenden Kriterien</u> muss <u>für mind. vier Stunden oder länger</u> erfüllt sein:

- mittlerer arterieller Druck (MAD) < 65 mmHg (< 8,7 kPa)
  oder
- systolischer arterieller Druck (SAD) < 90 mmHg (< 12 kPa)
  oder
- der Einsatz von Vasopressoren zur Aufrechterhaltung:
  - **des SAD ≥ 90 mmHg (≥ 12 kPa) oder**
  - des MAD ≥ 65 mmHg (≥ 8,7 kPa).

<u>Definition adäquater Volumenstatus</u>: zentralvenöser Druck (ZVD) ≥ 8 mmHg ohne maschinelle Beatmung oder ZVD ≥ 12 mmHg mit maschineller Beatmung **und** zentralvenöse Sauerstoffsättigung (ScvO2) ≥ 70 %. *Die zentralvenöse Sättigung wird in der Regel nur sehr selten bestimmt, da der Patient dafür einen Pulmonalarterienkatheter benötigt, der kaum noch verwendet wird.*

<u>Definition Vasopressoren</u>: Dopamin ≥ 5 µg/kg/min <u>oder</u> jede Dosis von Adrenalin, Noradrenalin, Vasopressin oder andere Vasopressoren. In der Regel werden nur die im CRF aufgeführten Vasopressoren verwendet.

<u>Die Kriterien des septischen Schocks sind **NICHT** erfüllt, wenn</u>:

- Patienten nur vorübergehend Vasopressoren erhalten (z.B. initiale Volumengabe, Intubation, Anästhesie) und sie danach für mindestens 2 Stunden vasopressorfrei und nicht hypotensiv sind
- Vasopressoren nur kurzfristig in geringer Dosierung verwendet werden, z.B. bei Lagerung, Transporten, Intubation, iatrogenen Maßnahmen etc.
- Hypotension offensichtlich nicht durch eine Sepsis bedingt ist, z.B. perioperativer Flüssigkeitsverlust, Applikation von Anästhetika, Blutung, etc.
- Sepsis-induzierter Volumenmangel besteht und die Hypotension durch Applikation durch Volumen behoben werden kann.
- Vasopressoren für weniger als 4 Stunden kontinuierlich appliziert werden.

Im Rahmen des ADAMON-Audits ist das Auftreten eines septischen Schocks nur bis Tag 14 zu berücksichtigen. Dabei muss anhand der Patientenakte überprüft werden, ob Anzeichen für einen septischen Schock vorliegen und dies korrekt im CRF erfasst wurde bzw. ob eine etwaige Angabe „septischer Schock“ im CRF sich anhand der Quelldaten bestätigen lässt.

**Primärer Endpunkt**<br>**CRF Seite: Tägliche Erhebung V_03, FU28_01**<br><u>Hinweis</u>: Wenn Patient vor Tag 14 von der ITS entlassen wurde **UND** externe Informationen nicht detailliert dokumentiert vorliegen, dann ist Kriterium nach ITS-Entlassung nicht beurteilbar.	**kritisch oder schwerwiegend**	**geringfügig**
Mindestens ein Kriterium für septischen Schock laut Quelldaten erfüllt <u>aber</u> septischer Schock nicht im CRF dokumentiert	X	
Septischer Schock im CRF dokumentiert <u>aber</u> anhand der Quelldaten sind Kriterien nicht nachvollziehbar	X	
Falls zutreffend: Beginn (Datum) septischer Schock in Quelldaten und CRF abweichend	X	
Sonstiges Problem (Beschreibung notwendig)		

5.6.5 Mängel bei Bestimmung der sekundären Endpunkte

Die folgenden sekundären Endpunkte sind für den Vergleich der beiden Therapiestrategien in der Hypress-Studie von wesentlicher Relevanz, und müssen daher beim Audit überprüft werden:

- 28-Tage Mortalität ausgehend von der Randomisation
- Liegedauer auf ITS
- Dauer der Beatmung bis ITS-Entlassung
- Dauer einer Nierenersatztherapie bis zur ITS-Entlassung
- Häufigkeit von weaning failure innerhalb von 28 Tagen/bis ITS-Enlassung
- Häufigkeit einer gastrointestinalen Blutung innerhalb von 28 Tagen/bis ITS-Enlassung

Folgende Hinweise sind bei der Überprüfung der sekundären Endpunkte zu beachten:

<u>Beatmung</u>
Jede Form der assistierten oder kontrollierten Beatmung gilt als Beatmung. Lediglich CPAP-Anwendungen zur Atemtherapie (z.B. CPAP einmalig pro Schicht zur Atelektasenprophylaxe) zählen nicht als Beatmung.

Eine Beatmung im Rahmen einer Operation ist nicht zu dokumentieren. Kann der Patient jedoch nicht extubiert werden, und es ergibt sich daraus eine verlängerte Beatmungsphase, sind die Beatmungsparameter zu dokumentieren.

<u>Weaning Failure</u>
→definiert als Reintubation innerhalb von 24 Stunden nach der Extubation oder nicht-invasive Beatmung mit Druckunterstützung für mehr als 48 Stunden nach Extubation. Die geplante Reintubation für eine geplante OP oder eine Bronchoskopie etc. ist kein AE.

<u>Gastrointestinale Blutung</u>
→ die, die Gabe von mindestens 2 Einheiten Erythrozytenkonzentraten innerhalb von 24 Stunden erfordert.

Sekundäre Endpunkte	kritisch oder schwerwiegend	geringfügig
28-Tage Mortalität<br>**CRF Seiten: IE_01, IE_02, F28_01**		
Es sind keine ausreichenden Quelldaten vorhanden, um zu entscheiden, ob der Patient innerhalb von 28 Tagen verstorben ist.<br>Hinweis: Wenn Patient vor Tag 28 von der ITS entlassen wurde, muss anhand der Akte nachvollziehbar sein, dass der Patient/die Angehörigen oder die weiterbehandelnde Einrichtung kontaktiert und zur Mortalität befragt wurden.	X	
Angaben zur Mortalität zum Zeitpunkt Tag 28 bzw. zum Tod zu früherem Zeitpunkt sind in CRF und Quelldaten abweichend	X	
Falls zutreffend: Angabe zum Todesdatum in Quelldaten und CRF abweichend	X	
Liegedauer auf ITS (ab ITS-Aufnahme/bis ITS-Entlassung)<br>**CRF Seiten: B_01, IE_01, IE_02, F28_01, FITS_01**		
Angaben zu Liegedauer in CRF und Quelldaten sind abweichend, und es ergibt sich eine Abweichung der Gesamt-Liegedauer um > 1 Tag	X	
Dauer der Beatmung bis ITS-Entlassung (ab Randomisation)<br>**CRF Seiten: Tägliche Erhebung V_12, F28_02, FITS_01**		
Angaben zur Dauer der Beatmung in CRF und Quelldaten sind abweichend, und es ergibt sich eine Abweichung der Gesamt-Beatmungsdauer um > 1 Tag	X	
Dauer einer Nierenersatztherapie bis zur ITS-Entlassung (ab Randomisation)<br>**CRF Seiten: Tägliche Erhebung V_13, F28_02, FITS_01**		
Angaben zur Dauer der Nierenersatztherapie in CRF und Quelldaten sind abweichend, und es ergibt sich eine Abweichung der Gesamt-Dauer der Nierenersatztherapie um > 1 Tag	X	
Weaning failure innerhalb von 28 Tagen/bis ITS-Entlassung (ab Randomisation)<br>**CRF Seiten: Tägliche Erhebung V_12, F28_02, AE**		
Angaben zum Ereignis in CRF und Quelldaten abweichend<br>Hinweis: Wenn Patient vor Tag 28 von der ITS entlassen wurde, dann ist Kriterium nur bis ITS Entlassung zu beurteilen.	X	
Gastrointestinale Blutung innerhalb von 28 Tagen/bis ITS-Entlassung (ab Randomisation)<br>**CRF Seiten: Tägliche Erhebung, AE**		
Angaben zum Ereignis in CRF und Quelldaten abweichend<br>Hinweis: Wenn Patient vor Tag 28 von der ITS entlassen wurde, dann ist Kriterium nur bis ITS Entlassung zu beurteilen.	X	
Sonstiges Problem zu den sekundären Endpunkten (Beschreibung notwendig)		

5.6.6 Mängel bei der Erfassung und Meldung unerwünschter Ereignisse

Unerwünschte Ereignisse (egal ob erwartet oder unerwartet), die bis zum Tag 28 auftraten, waren in der Studiendatenbank (Event AE, Bogen AE) und falls zutreffend auch auf dem SAE-CRF (papierbasiert) zu dokumentieren.

Folgende sepsisrelevante Ereignisse werden als klinische Ereignisse im CRF erfasst und müssen nur dann als AE bzw. SAE dokumentiert werden, wenn ein kausaler Zusammenhang mit der Gabe der Prüfmedikation vermutet wird:

- Tod durch schwere Sepsis/septischer Schock
- Entwicklung eines septischen Schocks
- SIRS-Kriterien und Vitalparameter:
  - Fieber (≥38 °C) oder Hypothermie (≤ 36 °C)
  - Tachykardie (≥ 90/min) oder Bradykardie ≤ 50/min
  - Tachypnoe (≥ 20/min) oder Hyperventilation ($PaCO2$ ≤ 32 mmHg, ≤ 4,4 kPa) oder maschinelle Beatmung oder Dyspnoe

  - Leukozytose (≥ 12,000/µl) oder Leukopenie (≤ 4,000/µl) oder ≥ 10% unreife Granulozyten
- Hepathische Dysfunktion (Anstieg von Leberenzymen oder Bilirubin über den Normalwert bzw. Anstieg im Vergleich zum Ausgangswert)
- Renale Dysfunktion
  - Oligurie: ≤ 0,5 ml/kg/Std. über mindestens 2 Stunden trotz ausreichender Volumensubstitution und/oder
  - Anstieg von Harnstoff und / oder Kreatinin > 1,5 x Ausgangswert (Baselinewert) und / oder
  - Nierenersatztherapie wegen Sepsis-induziertem Nierenversagen
- Septische Enzephalopathie
  - Vigilanzstörung (Oberbegriff, dazu gehört auch zunehmende Bewusstseinstrübung bis hin zum Koma, Somnolenz)
  - Delir (achten auf psychomotorische Unruhe, Verwirrtheit, aber auch verwirrtes Denken, Fehldeutung der Umgebung, Angstzustände, Aggressionen, Desorientiertheit)
  - psychomotorische Unruhe
- Hämostatische Dysfunktion
  - Thrombozytopenie (< 10000/µl bzw. < 10/nl bzw. < 10 G/l)
  - Thrombozytose (> 400000/µl bzw. > 400 /nl bzw. > 400 G/l)
  - DIC (Zeichen für eine DIC können sein: Blutungsneigung, Anstieg von D-Dimeren oder Fibrinspaltprodukten im Vergleich zum Ausgangswert (Baseline) (wird nicht immer bestimmt), Abfall von Quick, Thrombozyten; Verbrauch von Gerinnungsfaktoren wie Fibrinogen, AT III (wird nicht immer bestimmt). Achten auf therapeutische Massnahmen , die auf eine DIC hinweisen könnten: Gabe von Frischplasma, Thrombozytenkonzentrate, EK bei Blutungen)
  - Koagulopathie: Thrombozyten ≤ 100000/µl oder > 30 % Abfall innerhalb von 24 Stunden; die Thrombozytopenie darf nicht hämorrhagisch oder immunologisch bedingt sein
  - Veränderung von Gerinnungsparametern Abfall von bei Gerinnungsparametern wie Quick, aPPT, Thrombozyten, Fibrinogen, Anstieg von D-Dimeren, Monomeren, Finbrinspaltprodukten > 50 % vom Ausgangswert
- Respiratorische Dysfunktion:
  - Hypoxie (PaO_2 ≤ 75 mmHg (≤ 10 kPa) bei Raumluft)
  - ALI/ARDS (PaO_2/FiO_2 ≤ 250 mmHg (≤ 40 kPa) unter O_2-Applikation)
  - Beatmung (jede neu initiierte maschinelle oder nicht-invasive Beatmung kann auf respiratorische Insuffizienz hinweisen, auf andere Indikationen wie Nachbeatmung nach OP achten)
  - Abfall von PaO_2/FiO_2: achten auf Abfall um > 30 %.
  - Die Hypoxämie darf nicht durch kardiale oder pulmonale Ursache bedingt sein (z.B. Emphysem)
  - weitere Ursachen für respiratorische Insuffizienz: Pneumothorax, Lungenödem, Hämatothorax, kardiale Insuffizienz, Exazerbation vorbestehender chronischer pulmonaler Erkrankung, Lungenembolie u.a.
- Metabolische und mikrozirkulatorische Dysfunktion:
  - Hypokalämie (< 3.0 mmol/l bzw. 12 mg/dl)
  - Hyperkalämie (> 6 mmol/l bzw. 24 mg/dl)
  - Akrozyanose (Akrozyanose betrifft Finger und Füße bzw. auch Marmorierung, diese findet sich aber auch an anderen Hautarealen)
  - Laktatanstieg: > 1,5 x vom Ausgangswert und/oder
  - metabolische oder respiratorische Änderungen im Säure-Basen-Haushalt: Basendefizit ≥ 5 mmol/l und/oder metabolische Azidose mit pH < 7,3 und /oder
  - gestörte kapillare Reperfusion und/oder
  - signifikante Ödeme bei Kapillarlecksyndrom.
- Notfall-OP

***Hinweis:* Die oben genannten sepsisrelevanten Ereignisse sind im Rahmen des Audits nur dann zu überprüfen, falls eine SAE-Meldung erforderlich war. Hierbei sind die Angaben auf dem SAE Formular mit der Patientenakte abzugleichen.**

Die folgenden möglichen substanzspezifischen Nebenwirkungen des Prüfproduktes, die für die Fragestellung der Studie von speziellem Interesse sind, **oder** die nicht sepsis-typischen Ereignisse waren bis Tag 28 als AE bzw. SAE zu dokumentieren, unabhängig davon, ob ein kausaler Zusammenhang mit der Gabe des Prüfproduktes vermutet wurde.

Substanzspezifische Nebenwirkungen:

- Weaning Failure

- Gastrointestinale Blutung
- Sekundäre Infektion → ***Da es im Ermessen des behandelnden Arztes liegt, zu beurteilen, ob eine Infektion bereits primär bestand oder sekundär erworben wurde, ist dieses Kriterium beim Audit nur zu überprüfen, falls Hinweise auf ein SAE vorliegen.***
- Muskelschwäche → ***Da der MRC Score nicht bei allen Patienten sondern in der Regel nur bei kooperativen und wachen Patienten erhoben wurde, ist dieses Kriterium bei keinem Patienten zu auditieren.***

Hinweis: Die substanzspezifischen Nebenwirkungen (Weaning Failure und Gastrointestinale Blutung) werden bereits im Zusammenhang mit den sekundären Endpunkten auditiert. Darüber hinaus sind diese Nebenwirkungen nur dann zu überprüfen, falls eine SAE Meldung erforderlich war.

Zu den nicht "sepsis-typischen" Ereignisse zählen z.B.:

- Myokardinfarkt
- Therapeutisch relevante Herzrhythmusstörungen
- Apoplex
- Lungenembolie etc.

Bei der folgenden Beurteilung der Mängel sind nur Ereignisse zu berücksichtigen, die mindestens ein SAE Kriterium erfüllen. SAEs waren vom Studienbeginn bis zum Tag 28 zu dokumentieren und innerhalb von 24h nach Kenntnisnahme vom Prüfzentrum an den Sponsorbevollmächtigten zu faxen. Beim Audit werden Meldungen innerhalb von 3 Tagen nach Kenntnisnahme des SAEs akzeptiert. Sollte eine Verlängerung der Hospitalisierung eine SAE Meldung erforderlich machen, muss anhand der Akte nachvollziehbar sein, dass die Dauer des Krankenhausaufenthaltes auf das Ereignis zurückzuführen ist und somit von der üblichen Aufenthaltsdauer abweicht.

**Erfassung und Meldung von SAEs**	**kritisch oder schwerwiegend**	**geringfügig**
Ein aus den Quelldaten ersichtliches SAE wurde nicht gemeldet.	X	
Die Quelldaten zu einem gemeldeten SAE fehlen oder sind nicht lesbar oder sind nicht eindeutig interpretierbar.	X	
SAE (Initial Report) wurde nicht prüfplangerecht gemeldet.<br>Hinweis: Der Zeitpunkt der SAE-Meldung (Datum Eingang beim ZKS-Leipzig = date of receipt) wird anhand der SAE Listen überprüft.<br>Eine Meldung innerhalb von 3 Tagen nach Kenntnisnahme wird beim Audit akzeptiert.	X	
Vor 2. AMG-Änderungsgesetz<br>Die Unterschrift auf der SAE-Meldung fehlt oder stammt von einem Arzt für welchen keine zustimmende Bewertung durch die EK vorliegt, unabhängig von der Autorisierung auf der PDL durch den Hauptprüfer.	X	
Nach 2. AMG-Änderungsgesetz<br>Die Unterschrift auf der SAE-Meldung fehlt oder stammt von einem Arzt, der nicht auf der PDL durch Unterschrift des Prüfers autorisiert wurde.	X	
Angaben zu Patient-ID, Event (Event, Date and time of onset, Serious event) auf dem SAE Report sind abweichend von den Quelldaten.<br>Hinweis: Weitere Angaben zum Event (Intensity, Outcome, Causal relationship) sowie IMP, Medical history / Concomitant therapy, lab findings werden nicht auditiert.	X	
Geringfügige interpretationsbedingte Abweichungen hinsichtlich der Datumsangabe zum SAE.<br>Hinweis: Das gilt z.B. falls Start des AEs auf dem SAE Bogen dokumentiert wurde, obwohl das SAE Kriterium (Hospitalisierung) erst einen Tag später erfüllt war.		X
Sonstiges Problem (Beschreibung notwendig)		

5.6.7 Mängel bei der Nachbeobachtung

Die Follow-up Untersuchungen waren laut Prüfplan am Tag 28 bzw. Tag der Krankenhaus- Entlassung, Tag 90 und Tag 180 durchzuführen, um die Mortalität zu erfassen. Eine Überprüfung der Daten im Rahmen des Audits erfolgt nur bis zum Tag 28 und gehört somit zu den Auditkriterien „sekundäre Endpunkte“.

5.7 Erstellen der Auditberichte

Die Auditoren berichten die Durchführung des Audits mit den entsprechenden Formblättern (Audit CRF Allgemein, Audit CRF Patient, Auditbericht) möglichst innerhalb von 14 Arbeitstagen an den ADAMON Projektleiter. Nach Review der Dokumente durch die ADAMON Projektleitung, werden diese innerhalb von 14 Arbeitstagen zur Finalisierung an die Auditoren gesendet. Nach Finalisierung und Unterzeichnung der Dokumente gehen diese im Original an die ADAMON-Projektleitung.

Die Prüfzentren werden vor Ort im Rahmen eines Abschlussgespräches über die Ergebnisse des Audits informiert und erhalten im Anschluss eine Auditbestätigung. Die finalen Auditberichte aller Prüfzentren werden der Studienleitung elektronisch durch die ADAMON Projektleitung zur Verfügung gestellt.

6 Abkürzungsverzeichnis

AMG	Gesetz über den Verkehr mit Arzneimitteln – Arzneimittelgesetz
CRF	Case Report Form
DM	Datenmanagement
eDE	elektronische Dateneingabe
eRT	eResearch Technology
GCP	Good Clinical Practice
ISF	Investigator Site File
KKS	Koordinierungszentrum für Klinische Studien
LKP	Leiter der Klinischen Prüfung
PDL	Personal- und Delegierungsliste
SDV	Source Data Verification
Stud-PID	Studien-Patienten-Identifikation
TMF	Trial Master File
ZKS	Zentrum für Klinische Studien

7 Anhang

Hypress_Auditmanual_Prüfzentren

Briefvorlage zur Ankündigung der Audits

Vorlage zur Auditbestätigung

Audit CRF (Vorlage Patient, Vorlage Allgemein)

Auditbericht (Vorlage)

SAE Liste für alle Prüfzentren

Auflistung durch Ethikkommission zustimmend bewerteter Prüfer, Stellvertreter sowie gemeldete ärztliche Mitarbeiter

Patientenlisten einschließlich relevanter Zeitpunkte:

- Randomisierung, Therapiebeginn/-ende, Therapiedauer,
- Grund Therapieende

- Ende Dokumentation
- Medkit ID
- Entlassung ITS
- Angabe Gesamtdosis IMP: SOLL / IST / Toleranzbereich ±20%
- Datum Tag 14, Tag 28

Excel Tool zur Berechnung der Therapietage

Patientenbezogenes Audit - für jeden Patienten auszufüllen

Durchführbarkeit des Audits				
Sind folgende Kriterien zur Durchführung des Audits erfüllt? *GCP 8.3.13, 8.3.14*	Ja	Nein	N/A	Komm.
1) Liegen eine Akte und das eCRF des Patienten vor?<br>*<u>Hinweis</u>: Wenn Akte und/oder Zugang zum eCRF nicht existieren, ist dies als Finding zu werten.*	☐	☐	☐	☐
2) Liegt die Akte des Patienten vollständig vor?<br>*<u>Hinweis</u>: Falls nein, fehlende Dokumente bitte im Kommentarfeld auflisten.*	☐	☐	☐	☐
Bei Bedarf bitte kommentieren (max. 240 Zeichen).				

Art der Quelldokumente				
3) Welche Quelldokumente sind für den Patienten vorhanden?	Ja	Nein	N/A	Komm.
Papierakte	☐	☐	☐	☐
Elektronische Akte	☐	☐	☐	☐
Digitalisierte Akte	☐	☐	☐	☐

4) Auf welche Quelldokumente hat der Auditor Zugriff?	Ja	Nein	N/A	Komm.
Papierakte	☐	☐	☐	☐
Welche Dokumente gehören zur Akte (z.B. Stationskurve, OP-Bericht, Arztbrief, Röntgenbefund)? *(max. 240 Zeichen)*				
Elektronische Akte liegt als Papierausdruck vor	☐	☐	☐	☐
Welche Dokumente lagen als Papierausdruck vor (z.B. Stationskurve, OP-Bericht, Arztbrief, Röntgenbefund)? *(max. 240 Zeichen)*				
Elektronische Akte	☐	☐	☐	☐
Welche Dokumente gehören zur Akte <u>und</u> wie sind Eintragungen/ Änderungen nachverfolgbar (z.B. Audit Trail)? *(max. 240 Zeichen)*				
Digitalisierte Akte liegt als Papierausdruck vor	☐	☐	☐	☐
Welche Dokumente lagen als Papierausdruck vor (z.B. Stationskurve, OP-Bericht, Arztbrief, Röntgenbefund)? *(max. 240 Zeichen)*				
Digitalisierte Akte	☐	☐	☐	☐
Welche Dokumente gehören zur Akte (z.B. Stationskurve, OP-Bericht, Arztbrief, Röntgenbefund)? *(max. 240 Zeichen)*				

5) Falls <u>elektronische / digitalisierte Akte</u>, wie erfolgte Zugriff durch den Auditor, *bitte nachfolgend auflisten:*

Bei Bedarf bitte kommentieren (max. 240 Zeichen).

**EWE bei primär einwilligungsfähigen Patienten**				
	Ja	Nein	N/A	Komm.
6) Sind **schwerwiegende oder kritische** Mängel hinsichtlich Aufklärungsprozess und Einwilligungserklärung (EWE) aufgetreten?	☐	☐	☐	☐
Wenn ja, bitte zutreffende Punkte auswählen und kommentieren:				
7) EWE vom Patienten liegt nicht vor (weder Original noch Kopie). *GCP 8.3.12*	☐	☐	☐	☐
8) Nur die Unterschriftenseite der EWE liegt vor und Patient ist nicht identifizierbar.<br>Hinweis: Falls der Patient durch Namen oder Patienten-Nr. identifizierbar ist und die Unterschriftenseite korrekt datiert und unterschrieben wurde, ist dies als geringfügiger Mangel zu bewerten.	☐	☐	☐	☐
Mängel hinsichtlich Einwilligung durch Patient GCP 4.8.8				
9) Die Unterschrift des Patienten fehlt	☐	☐	☐	☐
10) Das eigenhändige Datum des Patienten fehlt<br>und es lässt sich nicht anhand eines zeitgleichen Eintrags in der Patientenakte/ NTF nachvollziehen, dass die Einwilligung vor Randomisation erfolgt ist.<br>Hinweis: falls anhand Akte/NTF nachvollziehbar oder Datum nachträglich vom Patienten durch Unterschrift bestätigt -> geringfügiger Mangel	☐	☐	☐	☐
11) Das eigenhändige Datum des Patienten liegt nach Randomisation<br>und es lässt sich nicht anhand eines zeitgleichen Eintrags in der Patientenakte nachvollziehen, dass die Einwilligung vor Randomisation erfolgt ist.<br>Hinweis: falls anhand Akte nachvollziehbar -> geringfügiger Mangel	☐	☐	☐	☐
Mängel hinsichtlich Bestätigung der mündlichen Einwilligung durch unabhängigen Zeugen GCP 4.8.9				
12) Unterschrift stammt von einem Zeugen, der zum Prüfteam (siehe PDL) oder Stationspersonal gehört	☐	☐	☐	☐
13) Unterschrift des unabhängigen Zeugen fehlt	☐	☐	☐	☐
14) Unabhängiger Zeuge hat EWE nicht eigenhändig datiert<br>und es lässt sich nicht anhand eines zeitgleichen Eintrags in der Patientenakte/ NTF nachvollziehen, dass die mündliche Einwilligung des Patienten vor Randomisation erfolgt ist.<br>Hinweis: falls anhand Akte/ NTF nachvollziehbar oder Datum nachträglich vom Patienten durch Unterschrift bestätigt -> geringfügiger Mangel	☐	☐	☐	☐
Mängel hinsichtlich Aufklärung durch Prüfer/qualifizierten ärztlichen Mitarbeiter GCP 4.8.8, 4.8.1				
15) Die Unterschrift des aufklärenden Prüfers/qualifizierten ärztlichen Mitglieds der Prüfgruppe fehlt.	☐	☐	☐	☐
Vor 2. AMG-Änderungsgesetz<br>16) Die Aufklärung/Unterschrift erfolgte durch einen Arzt, für welchen jedoch keine zustimmende Bewertung durch die EK vorliegt.<br>Hinweis: es spielt hier keine Rolle, ob der Arzt lt. PDL autorisiert war oder nicht	☐	☐	☐	☐
Nach 2. AMG-Änderungsgesetz<br>17) Die Aufklärung/Unterschrift erfolgte durch einen Arzt, der nicht durch den Prüfer auf der PDL dafür autorisiert wurde.	☐	☐	☐	☐

**EWE bei primär einwilligungsfähigen Patienten**				
	Ja	Nein	N/A	Komm.
18) Das Datum der Unterschrift des aufklärenden Prüfers/qualifizierten ärztlichen Mitglieds der Prüfgruppe fehlt *oder* liegt nach Randomisation<br>und es lässt sich nicht anhand des Datums der Einwilligung durch den Patienten nachvollziehen, dass die Aufklärung vor Randomisation stattgefunden hat.<br>Hinweis: falls anhand Akte od. Einwilligungsdatum nachvollziehbar-> geringfügiger Mangel	☐	☐	☐	☐
19) Sonstige **schwerwiegende oder kritische** Mängel	☐	☐	☐	☐
Wenn *Sonstige (max. 240 Zeichen)*, bitte beschreiben:				
Kommentare (max. 240 Zeichen) bitte angeben.				

**EWE bei nicht-einwilligungsfähigen Patienten** *GCP 4.8.15*				
	Ja	Nein	N/A	Komm.
20) Sind **schwerwiegende oder kritische** Mängel hinsichtlich Aufklärungsprozess und Einwilligungserklärung (EWE) aufgetreten?	☐	☐	☐	☐
Wenn ja, bitte zutreffende Punkte auswählen und kommentieren:				
21) Mutmaßlicher Patientenwille wurde nicht beachtet soweit zum Studieneinschluss bekannt	☐	☐	☐	☐
22) Vorhandensein einer Vorsorgevollmacht wurde nicht berücksichtigt obwohl zum Studieneinschluss bekannt	☐	☐	☐	☐
23) Vorhandensein eines Betreuungsverhältnisses wurde nicht berücksichtigt obwohl zum Studieneinschluss bekannt	☐	☐	☐	☐
24) EWE vom Betreuer/Bevollmächtigten liegt nicht vor *GCP 4.8.8*<br>Hinweis: Nur zutreffend, wenn Betreuer/Bevollmächtigter zum Studieneinschluss vorhanden oder Betreuer später bestellt wurde.	☐	☐	☐	☐
25) EWE durch vermeintlichen Betreuer/Bevollmächtigten erfolgt, aber Nachweis, wie Kopie der Vorsorgevollmacht oder Betreuungsurkunde, liegt nicht vor	☐	☐	☐	☐
26) EWE vom Betreuer/Bevollmächtigten ist Patient weder durch Namen noch durch Patienten-Nr. zuzuordnen.	☐	☐	☐	☐
Mängel hinsichtlich Einwilligung durch Betreuer/Bevollmächtigten GCP 4.8.8				
27) Die Unterschrift des Betreuers/Bevollmächtigten fehlt	☐	☐	☐	☐
28) Das eigenhändige Datum des Bevollmächtigten fehlt,<br>und es lässt sich nicht anhand eines zeitgleichen Eintrags in der Patientenakte/NTF nachvollziehen, dass die Einwilligung des Bevollmächtigten vor Randomisation erfolgt ist.<br>Hinweis: falls anhand Akte/NTF nachvollziehbar -> geringfügiger Mangel	☐	☐	☐	☐
29) Das eigenhändige Datum des Betreuers fehlt,<br>und es lässt sich nicht anhand eines zeitgleichen Eintrags in der Patientenakte/ NTF nachvollziehen, dass die Einwilligung nach Einrichtung des Betreuungsverhältnisses erfolgt ist.<br>Hinweis: falls anhand Akte/ NTF nachvollziehbar -> geringfügiger Mangel	☐	☐	☐	☐
30) Es existiert bereits vor Einschluss ein Betreuungsverhältnis bzw. es liegt eine Vorsorgevollmacht vor, das eigenhändige Datum des Betreuers/ Bevollmächtigten liegt trotzdem nach Randomisation<br>und es lässt sich nicht anhand eines zeitgleichen Eintrags in der Patientenakte nachvollziehen, dass die Einwilligung vor Randomisation erfolgt ist.<br>Hinweis: falls anhand Akte nachvollziehbar -> geringfügiger Mangel	☐	☐	☐	☐

**EWE bei nicht-einwilligungsfähigen Patienten** *GCP 4.8.15*				
	Ja	Nein	N/A	Komm.
Mängel hinsichtlich Einwilligung durch Patient				
31) *Falls zutreffend*: Nachträgliche Unterschrift des Patienten fehlt	☐	☐	☐	☐
Mängel hinsichtlich Erklärung Konsiliararzt, falls zutreffend				
32) Konsiliararzt nicht unabhängig von Prüfstelle<br>Hinweis: Der Konsiliararzt darf nicht an der Studie beteiligt sein, nicht zum Betreuungsteam des Patienten gehören und auch nicht der studiendurchführenden Station angehören. Die entsprechende Überprüfung erfolgt in der Regel anhand des Stempels auf der Konsiliararzterklärung.	☐	☐	☐	☐
33) Erklärung des Konsiliararztes über die Studienteilnahme liegt nicht vor	☐	☐	☐	☐
34) Konsiliararzterklärung ist dem Patienten weder durch Namen noch durch Patienten-Nr. zuzuordnen.	☐	☐	☐	☐
35) Konsiliararzt hat die Studienteilnahme nicht befürwortet, Patient wurde trotzdem in die Studie eingeschlossen	☐	☐	☐	☐
36) Datum der Konsiliarzterklärung liegt nach Randomisierung	☐	☐	☐	☐
37) Betreuungsverhältnis wurde nicht innerhalb von 3 Kalendertagen nach dem Tag der Randomisation beantragt und Patient ist nicht innerhalb von 3 Kalendertagen wieder einwilligungsfähig.<br>Hinweis: In der Akte muss die Beantragung belegt sein, z.B. Fax an Amtsgericht.	☐	☐	☐	☐
38) Sonstige **schwerwiegende oder kritische** Mängel	☐	☐	☐	☐
Wenn *Sonstige* *(max. 240 Zeichen)*, bitte beschreiben:				
Kommentare (max. 240 Zeichen) bitte angeben.				

**Datenschutzerklärung (DSE)**<br>*Bei Patienten, die primär nicht einwilligungsfähig waren, ist eine Überprüfung der nachträglichen Unterschrift des Patienten auf der DSE im Rahmen des Audits nur dann erforderlich, falls die DSE primär nicht vom Betreuer/Bevollmächtigten unterschrieben wurde.*				
	Ja	Nein	N/A	Komm.
39) Sind **schwerwiegende oder kritische** Mängel hinsichtlich der Datenschutzerklärung (DSE) aufgetreten?	☐	☐	☐	☐
Wenn ja, bitte zutreffende Punkte auswählen und kommentieren:				
40) DSE liegt nicht vor. (weder Original noch Kopie). *GCP 8.3.12*	☐	☐	☐	☐
41) DSE ist dem Patienten nicht zuzuordnen weder durch Namen noch durch Patienten-Nr. oder als Anhang zur EWE.	☐	☐	☐	☐
42) Die Unterschrift des Patienten/Betreuers/Bevollmächtigten fehlt. *GCP 4.8.10*	☐	☐	☐	☐
43) Sonstige **schwerwiegende oder kritische** Mängel	☐	☐	☐	☐
Wenn *Sonstige* *(max. 240 Zeichen)*, bitte beschreiben:				
Kommentare (max. 240 Zeichen) bitte angeben.				

Einschlusskriterien				
	Ja	Nein	N/A	Komm.
44) Sind **schwerwiegende oder kritische** Mängel hinsichtlich der Einschlusskriterien aufgetreten? *GCP 4.5, 4.9.1, 4.9.2, 4.9.3*	☐	☐	☐	☐
Wenn ja, bitte zutreffende Punkte auswählen und kommentieren:				
45) **Kein klinischer Nachweis einer *INFEKTION***<br>***d.h. keines der unten aufgeführten Kriterien trifft zu***<br>Hinweis: Mindestens **1** Kriterium zum Nachweis einer Infektion erforderlich:<br>**a.** Nachweis pathologischer Mikroorganismen in Blut, Sputum, Urin oder normalerweise sterilem Körpergewebe<br>**b.** Identifizierbarer Fokus (z.B. purulentes Sputum oder Wundsekret, Darmperforation)<br>**c.** Nachweis von Granulozyten in normalerweise sterilem Gewebe<br>**d.** Klinisch vermutete Infektion ohne Nachweis pathogener Mikroorganismen (z.B. neues pulmonales Infiltrat im Rö-Thorax, behandelte Pneumonie, Purpura fulminans, nekrotisierende Fasciitis)	☐	☐	☐	☐
46) **Kein Nachweis von *SIRS* -** Systemisches inflammatorisches Response-Syndrom<br>***d.h. nur maximal eines der unten aufgeführten Kriterien trifft zu***<br>Hinweis: Mindestens **2** Kriterien zum Nachweis von SIRS erforderlich:<br>**a.** Fieber (≥38 °C) oder Hypothermie (≤ 36 °C)<br>**b.** Tachykardie (≥ 90/min)<br>**c.** Tachypnoe (≥ 20/min) oder Hyperventilation (PaCO2 ≤ 32mmHg, ≤ 4,4 kPa) oder maschinelle Beatmung<br>**d.** Leukozytose (≥ 12,000/µl) oder Leukopenie (≤ 4,000/µl) oder ≥ 10% unreife Granulozyten	☐	☐	☐	☐
47) **Kein Nachweis der *ORGANDYSFUNKTION***<br>***d.h. keines der unten aufgeführten Kriterien trifft zu***<br>Hinweis: Mindestens **1** Kriterium zum Nachweis der Organdysfunktion erforderlich:<br>**a. Enzephalopathie** ohne Beeinflussung durch psychotrope Pharmaka: Vigilanzstörung, Unruhe, Delirium, Desorientiertheit<br>**b. Akute renale Dysfunktion:** Urinausscheidung Oligurie ≤ 0,5 ml/kg/Std. über mindestens 2 Stunden trotz ausreichender Volumensubstitution und/oder Kreatinin-Anstieg > 2 x des oberen Normalwertes und/oder Nierenersatztherapie wegen Sepsis-induziertem Nierenversagen<br>**c. Koagulopathie:** Thrombozyten ≤ 100,000/µl oder > 30 % Abfall innerhalb von 24 Stunden; Die Thrombozytopenie darf nicht hämorrhagisch oder immunologisch bedingt sein.<br>**d. Pulmonale Dysfunktion/Hypoxämie:** PaO2 ≤ 75 mmHg (≤ 10 kPa) bei Raumluft oder PaO2/FiO2 ≤ 250 mmHg (≤ 33 kPa) unter O2-Applikation. Die Hypoxämie darf nicht durch kardiale oder pulmonale Ursache bedingt sein (z.B. Emphysem).<br>**e. Mirkrozirkulatorische Dysfunktion:** Laktat > 1,5 x des oberen Normalwertes und/oder Basendefizit ≥ 5 mmol/l und/oder metabolische Azidose mit pH < 7,3 und /oder gestörte kapillare Reperfusion und/oder Marmorierung der Haut und/oder signifikante Ödeme bei Kapillarlecksyndrom.	☐	☐	☐	☐
48) ***ORGANDYSFUNKTION* ≥ 48 Stunden**	☐	☐	☐	☐
49) Sonstige **schwerwiegende oder kritische** Mängel	☐	☐	☐	☐
Wenn *Sonstige (max. 240 Zeichen)*, bitte beschreiben:				

Einschlusskriterien				
	Ja	Nein	N/A	Komm.
Kommentare (max. 240 Zeichen) bitte angeben.				

Ausschlusskriterien				
	Ja	Nein	N/A	Komm.
50) Sind **schwerwiegende oder kritische** Mängel hinsichtlich der Ausschlusskriterien aufgetreten? *GCP 4.5, 4.9.1, 4.9.2, 4.9.3*	☐	☐	☐	☐
Wenn ja, bitte zutreffende Punkte auswählen und kommentieren:				
51) **Sepsis-induzierte *HYPOTENSION*** trotz adäquatem Volumenstatus, wenn eines der Kriterien für <u>mindestens 4 h</u> erfüllt ist:<br>- mittlerer arterieller Druck (MAD) < 65 mmHg (< 8,7 kPa)<br>***oder***<br>- systolischer arterieller Druck (SAD) < 90 mmHg (< 12 kPa)<br>***oder***<br>- der Einsatz von Vasopressoren zur Aufrechterhaltung:<br>• **des SAD ≥ 90 mmHg (≥ 12 kPa)** oder<br>• des MAD ≥ 65 mmHg (≥ 8,7 kPa)<br><u>Der adäquate Volumenstatus</u> ist definiert als: zentravenöser Druck (ZVD) ≥ 8 mmHg ohne maschinelle Beatmung oder ZVD ≥ 12 mmHg mit maschineller Beatmung **und** zentralvenöse Sauerstoffsättigung (ScvO2) ≥ 70 %.<br><u>Zu den Vasopressoren gehören</u>: Dopamin ≥ 5 µg/kg/min <u>oder</u> jede Dosis von Adrenalin, Noradrenalin, Vasopressin oder andere Vasopressoren.<br>Patienten, die nur vorübergehend Vasopressoren (z.B. initiale Volumengabe, Intubation, Anästhesie) erhalten, befinden sich nicht im septischen Schock, wenn sie danach für mindestens 2 Stunden vasopressorfrei und nicht hypotensiv sind und können in die Studie eingeschlossen werden.	☐	☐	☐	☐
52) Patienten mit Glukokortikoid-Medikation und bei denen eine Fortsetzung der Medikation bzw. eine Substitutionstherapie indiziert ist (z.B. > 10 mg Prednisolon-Äquivalent pro Tag für mindestens 5 Tage innerhalb der letzten 3 Monate)<br><u>Hinweis</u>: Eine Therapie mit topischen und/oder inhalativen Glukokortikoiden ist kein Ausschlusskriterium, wenn keine Indikation für eine Weiterbehandlung mit systemischen Glucocorticoiden besteht.	☐	☐	☐	☐
53) Alter < 18 Jahre	☐	☐	☐	☐
Ausschlusskriterien **– nur zu berücksichtigen, falls entsprechende Hinweise in der Akte zu finden sind**				
54) Hinweis auf bekannte Unverträglichkeit gegen Hydrocortison-21-hydrogensuccinat, Natriummonohydrogenphosphat oder Mannitol (Placebo).	☐	☐	☐	☐
55) Hinweis auf andere Indikation für systemische Glukokortikoid-Therapie (z.B. Anaphylaxie, COPD, Asthma, Autoimmunerkrankungen)	☐	☐	☐	☐
56) Hinweis auf Therapielimitierung (DNR-Order)<br><u>Hinweis</u>: do not resuscitate **-** Die Information ist der Patientenverfügung zu entnehmen, soweit vorhanden.	☐	☐	☐	☐
57) Hinweis auf Schwangerschaft	☐	☐	☐	☐
58) Hinweis auf Teilnahme an einer anderen interventionellen klinischen Studie innerhalb der letzten 30 Tage	☐	☐	☐	☐
59) Sonstige **schwerwiegende oder kritische** Mängel	☐	☐	☐	☐
Wenn *Sonstige* *(max. 240 Zeichen)*, bitte beschreiben:				

Ausschlusskriterien				
	Ja	Nein	N/A	Komm.
Kommentare (max. 240 Zeichen) bitte angeben.				

Prüfplangemäße Therapie<br>**CRF Seite: IMP, IE_01**				
	Ja	Nein	N/A	Komm.
60) Sind **schwerwiegende oder kritische** Mängel hinsichtlich der prüfplangemäßen Therapie aufgetreten? *GCP 4.5, 4.9.1, 4.9.2, 4.9.3*	☐	☐	☐	☐
Wenn ja, bitte zutreffende Punkte auswählen und kommentieren:				
61) Beginn der ersten Infusion (Bolus) erfolgte vor Randomisierung oder ≥ 4h nach Randomisation	☐	☐	☐	☐
62) Therapiebeginn obwohl septischer Schock bestand<br>Hinweis: Definition septischer Schock siehe Abschnitt zum primären Endpunkt.	☐	☐	☐	☐
63) Kein Therapieabbruch im Studienverlauf nachdem Kriterien für septischen Schock für mindestens 4h erfüllt waren	☐	☐	☐	☐
64) Angabe zur MedKit-ID im CRF nicht übereinstimmend mit den Quelldaten des Patienten (Randofax und/oder Drug Accountability) bzw. nicht anhand der Quelldaten nachvollziehbar	☐	☐	☐	☐
65) Angaben zur verabreichten Gesamtdosis auf der ITS in Quelldaten und CRF abweichend und mindestens eine der beiden Gesamtdosen (CRF und/oder Quelldaten) weicht um ± 20% von den Vorgaben im Prüfplan ab.	☐	☐	☐	☐
66) Gesamtdosis auf der ITS ohne medizinischen Grund um ± 20% abweichend von den Vorgaben im Prüfplan<br>Hinweis: Falls die Angaben zu einzelnen Infusionen zwischen CRF und Quelldaten abweichen, ist auch die Auswirkung auf die insgesamt verabreichte Gesamtdosis des Prüfproduktes auf der ITS zu überprüfen.	☐	☐	☐	☐
67) Anzahl der Tage mit Prüfmedikation entspricht nicht dem Therapiezeitraum laut Prüfplan <u>und</u> Gesamtdosis um ± 20% abweichend <u>ohne</u> dass medizinische Gründe vorliegen.<br>Hinweis: Falls Anzahl der Therapietage abweichend, die Gesamtdosis aber der Vorgabe im Prüfplan entspricht → geringfügiger Mangel	☐	☐	☐	☐
68) Sonstige **schwerwiegende oder kritische** Mängel	☐	☐	☐	☐
Wenn *Sonstige* *(max. 240 Zeichen)*, bitte beschreiben:				
Kommentare (max. 240 Zeichen) bitte angeben.				

***Primärer Endpunkt* - septischer Schock innerhalb von 14 Tagen**

CRF Seite: Tägliche Erhebung V_03 sowie FU28_01 (falls ITS-Entlassung vor Tag 14)

Hinweis: Wenn Patient vor Tag 14 von der ITS entlassen wurde und externe Informationen nicht detailliert dokumentiert vorliegen, dann ist Kriterium beim Audit nicht beurteilbar. Dies ist mit ‘N/A‘ zu kennzeichnen und entsprechend zu kommentieren.

Sepsis-induzierte HYPOTENSION trotz adäquatem Volumenstatus. Mindestens eines der folgenden Kriterien muss für mind. vier Stunden oder länger erfüllt sein:

- mittlerer arterieller Druck (MAD) < 65 mmHg (< 8,7 kPa)
  oder
- systolischer arterieller Druck (SAD) < 90 mmHg (< 12 kPa)
  oder
- der Einsatz von Vasopressoren zur Aufrechterhaltung:
  - **des SAD ≥ 90 mmHg (≥ 12 kPa) oder**
  - des MAD ≥ 65 mmHg (≥ 8,7 kPa).

	Ja	Nein	N/A	Komm.
69) Sind **schwerwiegende oder kritische** Mängel hinsichtlich des primären Endpunktes aufgetreten? *GCP 4.5, 4.9.1, 4.9.2, 4.9.3*	☐	☐	☐	☐
Wenn ja, bitte zutreffenden Punkt auswählen und kommentieren:				
70) Mindestens ein Kriterium für septischen Schock laut Quelldaten erfüllt aber septischer Schock nicht im CRF dokumentiert	☐	☐	☐	☐
71) Septischer Schock im CRF dokumentiert aber anhand der Quelldaten sind Kriterien nicht nachvollziehbar	☐	☐	☐	☐
72) *Falls zutreffend:* Beginn (Datum) septischer Schock in Quelldaten und CRF abweichend	☐	☐	☐	☐
73) Sonstige **schwerwiegende oder kritische** Mängel	☐	☐	☐	☐
Wenn *Sonstige (max. 240 Zeichen)*, bitte beschreiben:				
Kommentare (max. 240 Zeichen) bitte angeben.				

Sekundäre Endpunkte

	Ja	Nein	N/A	Komm.
74) Sind **schwerwiegende oder kritische** Mängel hinsichtlich der sekundären Endpunkte aufgetreten? *GCP 4.5, 4.9.1, 4.9.2, 4.9.3*	☐	☐	☐	☐
Wenn ja, bitte zutreffenden Punkt auswählen und kommentieren:				
28-Tage Mortalität **CRF Seiten: IE_01, IE_02, F28_01**				
75) Es sind keine ausreichenden Quelldaten vorhanden, um zu entscheiden, ob der Patient innerhalb von 28 Tagen verstorben ist. Hinweis: Wenn Patient vor Tag 28 von der ITS entlassen wurde, muss anhand der Akte nachvollziehbar sein, dass der Patient/die Angehörigen oder die weiterbehandelnde Einrichtung kontaktiert und zur Mortalität befragt wurden.	☐	☐	☐	☐
76) Angaben zur Mortalität zum Zeitpunkt Tag 28 bzw. zum Tod zu früherem Zeitpunkt sind in CRF und Quelldaten abweichend	☐	☐	☐	☐
77) *Falls zutreffend:* Angabe zum Todesdatum in Quelldaten und CRF abweichend	☐	☐	☐	☐
Liegedauer auf ITS (ab ITS-Aufnahme/bis ITS-Entlassung) **CRF Seiten: B-01, IE_01, IE_02, F28_01, FITS_01**				
78) Angaben zu Liegedauer in CRF und Quelldaten sind abweichend, und es ergibt sich eine Abweichung der Gesamt-Liegedauer um > 1 Tag	☐	☐	☐	☐

Sekundäre Endpunkte				
Dauer der Beatmung bis ITS-Entlassung (ab Randomisation)<br>**CRF Seiten: Tägliche Erhebung V_12, F28_02, FITS_01**				
79) Angaben zur Dauer der Beatmung in CRF und Quelldaten sind abweichend, und es ergibt sich eine Abweichung der Gesamt-Beatmungsdauer um > 1 Tag	☐	☐	☐	☐
Dauer einer Nierenersatztherapie bis zur ITS-Entlassung (ab Randomisation)<br>**CRF Seiten: Tägliche Erhebung V_13, F28_02, FITS_01**				
80) Angaben zur Dauer der Nierenersatztherapie in CRF und Quelldaten sind abweichend, und es ergibt sich eine Abweichung der Gesamt-Dauer der Nierenersatztherapie um > 1 Tag	☐	☐	☐	☐
Weaning failure innerhalb von 28 Tagen/bis ITS-Entlassung (ab Randomisation)<br>**CRF Seiten: Tägliche Erhebung V_12, F28_02, AE**				
81) Angaben zum Ereignis in CRF und Quelldaten abweichend<br>Hinweis: Wenn Patient vor Tag 28 von der ITS entlassen wurde, dann ist Kriterium nur bis ITS-Entlassung zu beurteilen.	☐	☐	☐	☐
Gastrointestinale Blutung innerhalb von 28 Tagen/bis ITS-Entlassung (ab Randomisation)<br>Hinweis: Gabe von mindestens 2 Einheiten Erythrozytenkonzentraten innerhalb von 24 Stunden.<br>**CRF Seiten: Tägliche Erhebung, AE**				
82) Angaben zum Ereignis in CRF und Quelldaten abweichend<br>Hinweis: Wenn Patient vor Tag 28 von der ITS entlassen wurde, dann ist Kriterium nur bis ITS-Entlassung zu beurteilen.	☐	☐	☐	☐
83) Sonstige **schwerwiegende oder kritische** Mängel	☐	☐	☐	☐
Wenn *Sonstige (max. 240 Zeichen)*, bitte beschreiben:				
Kommentare (max. 240 Zeichen) und dazugehörigen Erhebungszeitpunkt bitte immer angeben.				

Erfassung und Meldung von SAEs				
	Ja	Nein	N/A	Komm.
84) Sind schwerwiegende oder kritische Mängel hinsichtlich der Erfassung und Meldung von SAEs aufgetreten? GCP 4.5, 4.9.1, 4.9.2, 4.9.3, 4.11.1	☐	☐	☐	☐
Wenn ja, bitte Zutreffendes auswählen:				
85) Ein aus den Quelldaten ersichtliches SAE wurde nicht gemeldet	☐	☐	☐	☐
86) Die Quelldaten zu einem gemeldeten SAE fehlen oder sind nicht lesbar oder sind nicht eindeutig interpretierbar.	☐	☐	☐	☐
87) SAE (Initial Report) wurde nicht prüfplangerecht gemeldet.<br>Hinweis: Der Zeitpunkt der SAE-Meldung (Datum Eingang beim ZKS-Leipzig = date of receipt) wird anhand der SAE Listen überprüft.<br>Eine Meldung innerhalb von 3 Tagen nach Kenntnisnahme wird beim Audit akzeptiert.	☐	☐	☐	☐
Vor 2. AMG-Änderungsgesetz<br>88) Die Unterschrift auf der SAE-Meldung fehlt oder stammt von einem Arzt für welchen keine zustimmende Bewertung durch die EK vorliegt, unabhängig von der Autorisierung auf der PDL durch den Hauptprüfer.	☐	☐	☐	☐
Nach 2. AMG-Änderungsgesetz<br>89) Die Unterschrift auf der SAE-Meldung fehlt oder stammt von einem Arzt, der nicht auf der PDL durch Unterschrift des Prüfers autorisiert wurde.	☐	☐	☐	☐

Erfassung und Meldung von SAEs				
90) Angaben zu Patient-ID, Event (Event, Date and time of onset, Serious event) auf dem SAE Report sind abweichend von den Quelldaten.<br>Hinweis: Weitere Angaben zum Event (Intensity, Outcome, Causal relationship) sowie IMP, Medical history / Concomitant therapy, lab findings werden nicht auditiert.	☐	☐	☐	☐
91) Sonstige **schwerwiegende oder kritische** Mängel	☐	☐	☐	☐
Wenn *Sonstige* *(max. 240 Zeichen)*, bitte beschreiben:				
Kommentare (max. 240 Zeichen) bei Bedarf bitte angeben.				

Sonstige Beobachtung *(Zusammenfassung relevanter geringfügiger Mängel)*

Datum

Unterschrift Auditor

Name in Druckbuchstaben
